# Integrated transcriptome and proteome analyses unveil cytoskeletal alterations in an endothelial model of monogenic diabetes

**DOI:** 10.1186/s13073-026-01615-z

**Published:** 2026-02-27

**Authors:** Dawid Skoczek, Damian Kloska, Marta Targosz-Korecka, Krzysztof Szade, Artur Biela, Jerzy Hohendorff, Marian Babincak, Aleksandra Kopacz, Maciej T. Malecki, Jacek Stepniewski, Neli Kachamakova-Trojanowska

**Affiliations:** 1 Malopolska Centre of Biotechnology, Jagiellonian University, Gronostajowa 7a str, Krakow, 30-387 Poland; 2 Doctoral School of Exact and Natural Sciences, Jagiellonian University, Lojasiewicza str 11, Krakow, 30-387 Poland; 3 Department of Physics of Nanostructures and Nanotechnology, Faculty of Physics, Astronomy and Applied Computer Science, Jagiellonian University, Lojasiewicza str 11, Krakow, 30-387 Poland; 4 Laboratory of Stem Cell Biology, Faculty of Biochemistry, Biophysics and Biotechnology, Jagiellonian University, Gronostajowa str 7, Krakow, 30-387 Poland; 5 National Synchrotron Radiation Centre SOLARIS, Jagiellonian University, Czerwone Maki str 98, Krakow, 30-392 Poland; 6 Department of Metabolic Diseases, Jagiellonian University Medical College, Jakubowskiego str 2, Krakow, 30-688 Poland; 7 Department of Medical Biotechnology, Faculty of Biochemistry, Biophysics and Biotechnology, Jagiellonian University, Gronostajowa str 7, Krakow, 30-387 Poland

**Keywords:** Diabetes, Disease modelling, hiPSCs, Endothelial dysfunction, Glycocalyx

## Abstract

**Background:**

HNF1A-MODY is a subtype of monogenic diabetes caused by mutations in the hepatocyte nuclear factor-1 homeobox A (HNF1A) gene. While the role of HNF1A in pancreatic beta cells has been extensively studied, its role in other cell types, such as endothelial cells (ECs), is less understood. Despite the pharmacologically controlled glycemia in HNF1A-MODY patients, still cardiovascular disorders and other endothelial dysfunction diseases, such as retinopathy, are quite common. Therefore, the aim of the current study was to look for potential molecular alterations in the ECs related to HNF1A-MODY disease.

**Methods:**

To understand the molecular pathways underlying this effect, ECs from three sets of human pluripotent stem cells (hiPSC-ECs) were used – two isogenic and one patient set. The patient set consisted of four hiPSCs lines with two healthy/control lines and two hiPSCs lines derived from HNF1A-MODY patients. The control isogenic set has a control (healthy) and two CRISPR/Cas9-mutated hiPSCs lines, with monoallelic or biallelic mutations in the *HNF1A*. The patient isogenic set consists of HNF1A-MODY patient hiPSCs line and two repaired (control) lines. All these lines were subsequently differentiated toward ECs (hiPSC-ECs) and used for global transcriptome, global proteome, and other functional analyses. Additionally, selected results were confirmed in primary ECs, where *HNF1A* expression was silenced.

**Results:**

The integrated global transcriptome and proteome analyses of the control isogenic set (mutated versus unmutated), show differences in actin-based cytoskeleton, general metabolism, and proteoglycan-related genes. All *HNF1A*-mutated hiPSC-ECs had shorter glycocalyx layer in comparison to their control counterparts. The same phenotype can be mimicked by silencing the *HNF1A* in primary ECs. Additionally, *HNF1A*-mutated control isogenic lines showed increased migratory potential, which aligns with a decrease in the actin stress fibres. Similarly, increased migration is observed after *HNF1A* silencing in patient-specific hiPSC-ECs or in primary ECs.

**Conclusions:**

Taken together, these results reveal for the first time that mutations in the *HNF1A* cause proteomic and transcriptomic changes in ECs that affect their function through reduction of the glycocalyx layer and changes in the cell migration. Cumulatively, these changes could account for signs of endothelial dysfunction in HNF1A-MODY patients.

**Supplementary Information:**

The online version contains supplementary material available at 10.1186/s13073-026-01615-z.

## Background

In 2021, one in ten people was affected by diabetes worldwide [1]. Even though most cases are associated with type 1 or type 2 diabetes (T1D, T2D), advances in molecular genetics are increasingly identifying new specific types of monogenic diabetes. One of these is maturity-onset diabetes of the young (MODY) [2]. MODY is a group of heterogeneous autosomal dominantly inherited diabetic disorders. It is usually diagnosed before the age of 25 and is characterized by non-insulin dependence and beta cell dysfunction that progresses with age [3]. To date, fourteen MODY subtypes have been reported. Each of them is directly caused by a mutation in a single MODY-related gene, and these patients are often misdiagnosed with T1D or T2D [3, 4]. Mutations in hepatocyte nuclear factor-1 homeobox A (HNF1A) account for the majority of MODY cases, and polymorphisms in this gene are found to be a risk factor for T2D [5].

HNF1A is a transcription factor that is highly expressed in the liver, kidney, intestine, and pancreatic islets [6]. It is a multi-domain protein, composed of an N-terminal dimerization domain (DD), a DNA-binding domain (DBD), and a C-terminal transactivation domain. In the pancreas it modulates islet functions, in the liver, it affects carbohydrate and lipid metabolism, while in the kidney it is responsible for maintaining glucose homeostasis [7]. The mutations in *HNF1A*, linked to MODY, are heterozygous, and scattered throughout the protein-coding region. It was confirmed that the type of the mutations may affect the functionality and stability of the HNF1A protein and the age at which HNF1A-MODY is diagnosed [8]. To model HNF1A-MODY human induced pluripotent stem cells (hiPSCs) are currently used. Differentiation of patient-specific or *HNF1A*-mutated hiPSCs to beta cells revealed alterations in mitochondrial function, glycolysis, calcium levels, glucose uptake, and insulin secretion [9–11]. When looking at the beta-cells transcriptome carrying a heterozygous mutation in *HNF1A*, pathway analysis revealed changes at core cellular pathways such as glucose metabolism and ATP production, gene transcription, intracellular protein transport (synthesis, ubiquitination, and exocytosis), cell stress response and cell signalling. Furthermore, other processes like cell cycle regulation and cell adhesion/motility were also altered in mutated islet cells. Interestingly, half of the genes differentially expressed in beta-cells were also changed in alpha-cells, suggesting that HNF1A dysfunction is a mutual effector in both cell types [9].

Although diabetes is a metabolic disease, it has pronounced effects on macro- and microvasculature, thus being the major cause of blindness, kidney failure, heart attacks, stroke, and lower limb amputation (WHO, 2022). In particular, the link between diabetes and an increased incidence of cardiovascular disease is well established. Interestingly, endothelial dysfunction often precedes the clinical diagnosis of T2D by several years [12]. Similarly, in HNF1A-MODY patients, microvascular complications, particularly retinopathy, were found to be common [13]. Despite a favourable lipid profile and decreased C-reactive protein (CRP) level described in these patients, they are characterized by an increased risk of cardiovascular complications as compared with non-diabetic family members [14]. It was also shown that HNF1A-MODY patients have abnormalities in endothelial function or/and the presence of an early atherosclerotic phenotype [15]. Recently, using a hiPSCs disease model, we have shown that in normoglycemic conditions, *HNF1A* mutations can cause an increase in endothelial permeability, which may be the foundation for subsequent vascular complications in HNF1A-MODY patients [16]. However, the molecular pathways causing endothelial leakage as well as other molecular changes affecting endothelial homeostasis in this diabetes subtype remain unknown. Therefore, looking for the molecular background of the endothelial dysfunction in HNF1A-MODY, in the current study, three different sets of hiPSC lines were used. The control isogenic set was based on a healthy donor hiPSCs line (control) and lines with a CRISPR/Cas9 introduced mutations in one or both alleles of the *HNF1A* gene within the DBD (previously described in [16]). The DBD domain is the domain in which mutations occur most frequently, and the most clinically severe cases of HNF1A-MODY are caused by mutations in this domain [17], therefore, the isogenic lines were engineered to harbour mutations within this domain (83–279 aa) of the *HNF1A*. The control isogenic set al.lows for a direct comparison of mutated versus unmutated cells of the same genetic background. The patient set consisted of hiPSCs lines from two different healthy donors and two HNF1A-MODY patient-derived lines with different heterozygous *HNF1A* mutations. Finally, an HNF1A-MODY patient isogenic hiPSCs set was generated, where patient cells with a heterozygous mutation in *HNF1A* were repaired through CRISPR/Cas9, thus obtaining two repaired isogenic control lines. By employing integrated analyses of the transcriptome and proteome, along with functional assays, a more comprehensive understanding of the molecular processes within the vasculature of HNF1A-MODY patients could be unveiled.

## Methods

### Generation of human-induced pluripotent stem cells (hiPSCs) lines

In the current study, three different hiPSCs sets of lines were used. The isogenic control-derived set of lines was based on a healthy donor hiPSCs line (control) in which monoallelic (MAC) or biallelic (BAC) mutations in the *HNF1A* gene were introduced through CRISPR/Cas9 (Additional file 1: Fig. S1). The lines were described in detail previously [16]. The second set (patient set), consisted of hiPSC lines from two different healthy donors (HD_1 and HD_2) and two HNF1A-MODY patient-derived lines (MP_1 and MP_2) (Additional file 1: Fig. S2). The third set represented an isogenic patient-derived panel, which included the original HNF1A-MODY patient line MP_1 alongside two CRISPR/Cas9-corrected clones generated from this parental line. This corrected line set is thoroughly described in Skoczek et al., 2026 (Human Genetics, resubmitted). Details on the *HNF1A* mutations in the specific lines/sets are shown in Table 1. All hiPSCs lines were derived from peripheral blood mononuclear cells (PBMCs). The blood collected from all human donors was processed after obtaining informed consent from the individuals and in agreement with the Jagiellonian University Bioethical Committee no. 122.6120.303.2016 (healthy donors) and 1072.6120.310.2018 (HNF1A-MODY patients). Subsequently, all PBMCs were reprogrammed using non-integrating Sendai vectors (Cytotune-iPS 2.0 Sendai Reprogramming kit, ThermoFisher Scientific, Waltham, MA, USA), according to the manufacturer’s protocol (PBMC feeder-free system). After approximately three weeks, hiPSCs colonies were picked and expanded. The pluripotency of all generated hiPSCs lines was confirmed (Additional file 1: Fig. S3, S4) as well as their spontaneous differentiation into all three germ layers (Additional file 1: Fig. S5) before being used for further experiments. Detailed methodology for these analyses is included in the supplementary figures section. All generated hiPSCs lines had normal karyotypes (Additional file 1: Fig. S6, S7) [18, 19].

**Table 1:** Description of the hiPSCs lines used in the current study

Isogenic control-derived set of lines		
Name of the line	Type of mutations and their localization in *HNF1A*	Impact of mutations on protein sequence
Control line (Control)	Biallelic wild type	N/A
Monoallelic (MAC)	c.(410_422del); (=) Monoallelic mutation (deletion) in Exon 2	Premature STOP codon in the mutated allele; DNA-binding (POU_S_) domain
Biallelic (BAC)	c.(401_413del); (401_413del) Biallelic mutation (deletion) in Exon 2	Premature STOP codon in both mutated alleles in the DNA-binding (POU_S_) domain

Patient set of lines		
Name of the line	Type of mutations and their localization in *HNF1A*	Impact of mutations on protein sequence
Healthy Donor-1 (HD_1)	Biallelic wild type	N/A
Healthy Donor-2 (HD_2)	Biallelic wild type	N/A
HNF1A-MODY (MODY3) Patient-1 (MP_1)	c.(235_236insG); (=) Monoallelic mutation (insertion) in Exon 1	p.(Glu79GlyfsX16); (=) Premature STOP codon in the mutated allele; linker region of the dimerization and DNA binding domain
HNF1A-MODY (MODY3) Patient-2 (MP_2)	c.(824 A > T);(=) Monoallelic mutation (substitution) in Exon 4	p.(Glu275Val); (=) Single amino acid substitution; DNA binding (POU_H_) domain

Isogenic patient-derived set of lines		
Name of the line	Type of mutations and their localization in *HNF1A*	Impact of mutations on protein sequence
Corrected clone 1	Biallelic wild type	N/A
Corrected clone 2	Biallelic wild type	N/A
HNF1A-MODY (MODY3) Patient-1 (MP_1)	c.(235_236insG); (=) Monoallelic mutation (insertion) in Exon 1	p.(Glu79GlyfsX16); (=) Premature STOP codon in the mutated allele; linker region of the dimerization and DNA binding domain

### Culture of hiPSCs

hiPSCs were grown on Geltrex-coated (Gibco) wells in Essential 8 Medium (E8, Gibco) at 37 °C in a humidified incubator at 5% CO_2_ (standard conditions). For subculturing, the hiPSCs were detached with 0.5 mM EDTA in PBS and then one-fifth was resuspended in a culture medium supplemented with 10 µM Y-27,632 (Focus Biomolecules) for 24 h. After that, the medium was changed to E8 alone and then was exchanged on a daily basis. The lack of mycoplasma contamination was routinely checked with PCR.

### Differentiation of hiPSCs into endothelial cells (hiPSC-ECs)

The hiPSCs were differentiated according to the protocol used in Kachamakova-Trojanowska et al., 2019 [16]. Briefly, 5 × 10^4^/well of hiPSCs were seeded on a Geltrex-coated 12-well plate and cultured for 3 days in standard conditions. On day zero, the medium was replaced with RPMI (Gibco) with a B27 supplement without insulin (Gibco) and 6 µM CHIR-99,021 (Focus Biomolecules). On day two, the medium was changed to RPMI with B27 supplement without insulin with 3 µM CHIR-99,021. On day four, the medium was changed to endothelial cell basal medium (EBM, PromoCell) with 50 ng/ml vascular endothelial growth factor (VEGF, StemCell Technology) and 10 ng/ml of basic fibroblast growth factor (bFGF, StemCell Technology). On day 7, differentiated hiPSC-ECs were purified by MoFlo cell sorter based on the CD31 marker and cultured to the first passage on fibronectin-coated wells in endothelial growth medium (EGM MV2, PromoCell) with additional supplementation of 15 ng/ml VEGF (complete medium). All experiments with hiPSC-ECs were conducted in the second week after the purification. The purified hiPSC-ECs had all tested markers of primary endothelial cells like CD31, VE-cadherin (Additional file 1: Fig. S8), Tie-2, VCAM-1 and showed functional similarity to primary cells, as previously described in Kachamakova-Trojanowska et al., 2019 [16].

### Culture of primary human aortic endothelial cells

For the gene silencing studies, we used human aortic endothelial cells (HAECs), which were provided by Gibco. The cells were grown in Endothelial Basal Medium (EBM-2) (Lonza) supplemented with EGM-2MV SingleQuot Kit Supplements & Growth Factors (Lonza) and 5% fetal bovine serum (FBS) (Lonza). Cells were cultured at 37 °C in a humidified incubator in 5% CO_2_ atmosphere. The cells used in all experiments were between passages 5 and 8. The lack of mycoplasma contamination was routinely checked with qPCR.

### Global transcriptome analysis

After differentiation, the hiPSC-ECs from the isogenic control-derived set of lines (control, MAC, and BAC), were detached with Accutase on day 16 or 17 of the differentiation, washed with PBS, and lysed with Fenozol. Cells from two independent differentiations in duplicate were used for subsequent RNA isolation using the Chomczynski method [20]. Then, Ion AmpliSeq Transcriptome Human Gene Expression Panel was used for the generation of the libraries. The obtained libraries were sequenced on Ion Proton™ Sequencing System and BAM files were mapped against the reference hg19_AmpliSeq_Transcriptome_ERCC_v1. The analysis of differentially expressed genes (DEGs) was done using DEseq-2 script [21] in the R environment. RNA-seq data have been deposited in the *BioStudies*,* Array Express* database at EMBL-EBI under accession number E-MTAB-10404. Additionally, the DEGs with adjusted *p*-value ≤ 0.105 and fold change above 1.5 were analysed for gene set enrichment (GSEA) with DAVID software [22], and results were visualized in the R environment. Global transcriptome analysis was also performed with the patient-derived isogenic set of lines (corrected clone 1, corrected clone 2, and MP_1). Cells from four independent differentiations (one sample/line) were collected on day 16 of the differentiation using Accutase, washed with PBS and lysed with Fenozol. RNA was isolated using the Chomczynski method [20]. The libraries were constructed using the Illumina Stranded mRNA Prep kit according to the manufacturer’s protocol. Samples were sequenced on the NextSeq 2000 using the NextSeq 1000/2000 P2 XLEAP-SBS Reagent Kit (100 Cycles) and a 2 × 51 bp read mode. The RNA alignment and gene expression quantification analysis were performed using the DRAGEN RNA v.4.2.7 for NextSeq 1000/2000. RNA-seq data have been deposited in the *BioStudies*,* ArrayExpress* database at EMBL-EBI under accession number E-MTAB-16548. Given that the DEseq-2 analysis for DEGs did not reveal that many significant genes, we performed Gene Set Enrichment Analysis (GSEA) using “ReactomePA”, “fgsea”, and “enrichplot” packages in R. Genes were ranked based on Wald statistic, and only significant pathways with p-adjusted and FDR q-value < 0.05 were used for further analysis. Clustering of the pathways was done with the function emapplot from “enrichplot” with default k-means clustering, based on the 100 most enriched pathways. The clusters were manually annotated based on representative pathways included in the cluster, that are presented in the ridgeplots.

### Mass spectrometry analysis

hiPSC-ECs (Control, MAC, and BAC) from four independent differentiations were seeded on a 6-well plate. Confluent cells were harvested to buffer containing 1% SDS in 0.1 M Tris (pH = 7.5). The protein samples (40 µg of protein) were processed with FASP (30 kDa cut-off). Proteins were reduced (50 mM dithiothreitol for 15 min) and alkylated (54 mM iodoacetamide for 20 min). The overnight incubation with trypsin at 37 °C was performed and peptides were collected. Mass spectrometry analysis of the peptide mixture was performed using the RSLCnano system connected online to the Orbitrap Q-Exactive system (Thermo Fisher Scientific). The survey scan was acquired with a resolution of 70,000 over a mass range of 300 to 2000 m/z with an automatic gain control (AGC) target of 1 × 10^6^. The MS/MS spectra were acquired with a resolution of 17,500 with an AGC target of 5 × 10^5^. The maximum ion accumulation times for the full MS and the MS/MS scans were 120 ms and 60 ms respectively. The lock mass option was used to perform internal calibration.

For data analysis, MaxQuant software (version 1.6.7.0) was used for search against the human database (SwissProt) using the Andromeda search engine. The search parameter settings include trypsin as a protease with two missed cleavages, oxidation of methionine, acetylation of protein N-terminus, and carbamidomethylation of cysteine as fixed modification. The mass spectrometry data were deposited to the ProteomeXchange Consortium with the dataset identifier PXD039471. Normalized relative label-free quantification data (LFQ) were log2 transformed and used for further analysis. For comparison of MAC and BAC to the control cells, Student’s t-test was used. All the results were then filtered with the threshold of 1.2-fold change. The differentially expressed proteins were analysed with enrichment analysis of gene ontology sets (GO) and pathways-based sets using ConsensusPathDB software [23].

### Migratory capacity – scratch assay

To assess the migratory capacity, 2.8 × 10^4^/well of hiPSC-ECs were seeded on Culture-Insert 2 Well in µ-Dish 35 mm (ibidi). After 24 h the silicone inserts were removed, and cells were washed with PBS and kept in an EGM-MV2 complete medium supplemented with 1 mM hydroxyurea (Sigma-Aldrich) to block the cells proliferation. Photos were taken at ten time points (every hour), including time just after the addition of hydroxyurea. The percentage of gap closure was calculated using ImageJ with the *Wound_healing_size tool plugin* [24].

### Immunofluorescence detection of glycocalyx components

To visualize glycocalyx 3 × 10^5^ of hiPSC-ECs were seeded on fibronectin-coated 10 mm coverslip 48 h before the experiment. After two days, cells were fixed with 4% paraformaldehyde (Sigma) at room temperature (RT) for 15 min. The cells were then washed three times with PBS and blocked with 3% bovine serum albumin (BSA) in PBS for 1 h at RT. To recognize sialic acid, cells were incubated overnight at 4 °C with biotinylated Sambucus Nigra Lectin (EBL, Vector Laboratories) in 3% BSA in PBS. On the next day, to visualize sialic acid, cells were washed three times with PBS and incubated with fluorophore-conjugated streptavidin at a dilution of 1:500 in 3% BSA. In the same step, the N-acetylglucosamine component of glycocalyx was stained with rhodamine-conjugated WGA lectin at a dilution of 1:500 for 60 min at RT. Nuclei were stained with DAPI (4′,6-diamidino-2-phenylindole) at a concentration of 10 µg/ml. After incubation, cells were washed for 5 minutes twice with PBS and once with distilled water. The coverslips were then mounted with mounting medium onto slide glass. High-resolution images were taken by LSM-510 confocal microscope (Carl Zeiss). The fluorescence intensity was quantified using ImageJ by the method of corrected total cell fluorescence (CTCF) with the formula CTCF = Integrated Density – (Area of selected cell x Mean fluorescence of background readings).

### Immunofluorescence staining of advanced glycation-end products (AGE)

To check the presence of AGE products 3 × 10^5^ of hiPSC-ECs were seeded on fibronectin-coated 10 mm coverslip 48 h prior to the experiment. After two days, cells were fixed with 4% paraformaldehyde (Sigma) at room temperature (RT) for 10 min. Then the cells were washed three times with PBS and incubated with blocking and permeabilization buffer containing 1% BSA, 10% normal goat serum and 0.3 M glycine in 0.1% PBS-Tween for 1 h at RT. After that, the cells were incubated with the I^o^ antibody (1:100, Abcam) in the same buffer overnight at 4 °C. Next day, cells were washed 3 times with PBS and incubated with secondary antibody (1:500) conjugated with Alexa Fluor 488 in 3% BSA in PBS for 1 h at RT. Nuclei were stained with DAPI (4′,6-diamidino-2-phenylindole) at a concentration of 10 µg/ml. After incubation, cells were washed for 5 minutes twice with PBS and once with distilled water. The coverslips were then mounted with mounting medium onto slide glass. High-resolution images were taken using an LSM-510 confocal microscope (Carl Zeiss). The fluorescence intensity was quantified using ImageJ by the method of corrected total cell fluorescence (CTCF) with the formula CTCF = Integrated Density – (Area of selected cell x Mean fluorescence of background readings).

### Atomic force microscopy (AFM) measurements

For AFM experiments, 4 × 10^5^ hiPSCs-ECs were seeded at a round glass coverslip, and then cells were grown for 48 h. Next, the sample with cells was gently mounted into the AFM liquid chamber (BioCell, JPKInstruments) and measured in Hanks’ Balanced Salt Solution (HBSS, Sigma-Aldrich) at the stable temperature of 36.2 °C. The AFM measurements were performed using a NanoWizard 3 NanoScience AFM (JPK Instruments) using spherical glass probes, with a radius of 1.25 μm, attached to a flexible cantilever with a spring constant of 0.02 N/m (Novascan). Measurements were conducted using a force mapping mode. For each cell, a spatial map of force vs. distance (FD) curves at a grid of 7 × 7 or 16 × 16 points were measured. The size of the grid corresponds to a square surface with dimensions from 10 μm × 10 μm to 25 μm × 25 μm. The resulting step size of approx. 1.5 μm was chosen to be comparable with the probe diameter. The position of scan areas was controlled by inverted optical microscopy (Olympus). Force-distance curves were measured at a speed of 2 μm/s. The contact time was 1 s, and the maximal applied force was 500 pN. Before and after a series of force-distance measurements, calibration of the cantilever spring constant was performed by using Nanowizard software. To determine the cell elastic modulus E and glycocalyx length L, we used a procedure proposed by Sokolov et al. [25]. The Hertz model was fitted to the part of the curve close to the maximal indentation, for which the glycocalyx is assumed to be almost squeezed. According to Alexander-de Gennes’ theory, the steric polymer brush model was fitted to the data to calculate the length of the glycocalyx layer. The parameters were derived using software written in Matlab environment.

To assess the contribution of actin stress fibres to cell stiffness, the elastic modulus and 2D maps of hiPSCs-ECs before and after treatment with 10 µM cytochalasin D (Merck) were determined using the force mapping mode. A Bruker MLCT-BIO-DC type C cantilever with a square pyramid probe was used for these measurements. As in previous AFM measurements, the sample with cells was gently mounted in the AFM liquid chamber (BioCell, JPK Instruments) and immersed in Hanks’ balanced salt solution (HBSS, Sigma-Aldrich) at a stable temperature of 36.2 °C. Force mapping measurements were then performed on randomly selected cells to determine the elastic modulus and to visualise the actin fibre structure. Cytochalasin D was then added directly to the HBSS solution in the AFM liquid chamber to give a final concentration of 10 µM. After 15 min, the AFM measurement was repeated. For each sample, before and after cytochalasin treatment, measurements were taken for at least 10 maps in the area of a square matrix of 40 × 40 μm, with a preserved scaling of 1 px /1 µm. A force-distance curve was plotted in each px. A constant indentation range, not exceeding 200 nm (approximately 10% of the cell height), was maintained throughout the measurements to avoid the influence of the hard substrate on the resulting elastic modulus values. The set of force-distance curves was analysed by fitting the Hertz-Sneddon model for a pyramidal indenter to determine the cell modulus E (which refers exclusively to the apparent Young’s modulus). All analyses were carried out using JPK data processing software.

### Transfection with small-interfering RNA

To perform gene silencing 24 h before transfection, 1.5 × 10^4^ cells per well were seeded in a 24-well plate. Transfections were performed with 30 nM siRNA targeted against the human *HNF1A* gene (Life Technologies, s529261) or 30 nM scrambled siRNA (Life Technologies, 4390846) using Lipofectamine™ RNAiMAX Transfection Reagent (Life Technologies) in Opti-MEM I Reduced Serum Medium (Life Technologies) according to the vendor’s instructions. The further analysis and treatment of cells were performed 72 h after transfection.

### Protein isolation and western blot analysis

The total protein was isolated using RIPA buffer (Sigma) with protease inhibitors (Roche). Protein concentration was determined by the BCA method (bicinchoninic acid method). 20 µg of protein were denatured and SDS-PAGE was performed, followed by wet electrotransfer to methanol-activated PVDF membranes (Amersham). The membranes were blocked in 5% skim milk (Millipore) in 0.1% Tween-TBS for 1 h and incubated overnight with primary antibodies against HNF1A (Abclonal AB3092), GAPDH (Santa Cruz Biotechnology sc-59540) at 4° C. Then they were washed with 0.1% Tween TBS and incubated for 1 h with HRP-linked secondary antibodies (Cell Signaling Technology, G21234). Visualization was performed using Millipore Western Substrate using ChemiDoc Imaging Systems (Bio-Rad).

### RNA isolation and real-time PCR

Total RNA was isolated using Total RNA Mini Kit (A&A Biotechnology), according to the manufacturer’s description. The RNA was eluted with 25 µL of nuclease-free water. Reverse transcription reaction was performed using High-Capacity cDNA Reverse Transcription Kit (Thermo Fisher Scientific, USA) according to the manufacturer’s protocol. Quantitative RT-PCR was conducted in a StepOnePlus real-time PCR system (Applied Biosystems) in a mixture containing SYBR Green PCR Master Mix (Sigma), specific primers (listed in Table 2) and cDNA. Primers were designed using the NCBI primer design tool “Primer-BLAST.” The *EEF2* housekeeping gene was used as a reference. Quantitative analysis of gene expression was carried out using the ΔΔCt protocol.

**Table 2:** List of the primer sequences used in the current study

Gene	Forward primer	Reverse primer
*EEF2*	TGAGCACACTGGCATAGAGGC	GACATCACCAAGGGTGTGCAG
*HNF1A*	GGCAAACGCAACCCACG	TCTGCAGCTGGCTCAGTTTAG
*IL1B*	GATGTCTGGTCCATATGAACTG	TTGGGATCTACACTCTCCAGC
*IL6*	AGACAGCCACTCACCTCTTCAG	TTCTGCCAGTGCCTCTTTGCTG
*COL1A1*	CGAAGACATCCCACCAATCAC	GTCACAGATCACGTCATCGC
*COL3A1*	TGGTCTGCAAGGAATGCCTGGA	TCTTTCCCTGGGACACCATCAG

### Enzyme-linked immunosorbent assays (ELISA)

7,5 × 10^4^ cells per well were seeded in a 12-well plate. On the next day, the medium was replaced and 24 h later, the medium was collected. IL-8 protein concentration was determined in a culture medium using Human IL-8/CXCL8 DuoSet ELISA (Bio-Techne) according to the manufacturer’s protocol.

### Visualization of the actin cytoskeleton

To visualize the actin cytoskeleton, 3 × 10^5^ hiPSC-ECs or HAEC were seeded on a 10 mm fibronectin-coated coverslip 48 h before the experiment. After two days, cells were fixed with 4% paraformaldehyde (Sigma) at room temperature (RT) for 15 min. To disturb actin stress fibres, five minutes before the fixation cells were treated with 10 µM cytochalasin D (Merck). The cells were then washed three times with PBS and permeabilized with 0.5% Triton-PBS solution for 15 min at RT. The cells were then blocked with 3% bovine serum albumin (BSA) in PBS for 1 h at RT. The cells were then incubated overnight at 4 ° C with Rhodamine Fluorescent Phalloidin (Abcam) in 3% BSA in PBS. The next day, the nuclei were stained with DAPI at a concentration of 10 µg/ml in PBS for 5 min in RT. After incubation, cells were washed for 5 minutes twice with PBS and once with distilled water. The coverslips were then mounted with mounting medium onto slide glass. High-resolution images were taken using an LSM-510 confocal microscope (Carl Zeiss) or a fluorescent microscope Leica DMi8. The fluorescence intensity was quantified using ImageJ by the method of corrected total cell fluorescence (CTCF) with the formula CTCF = Integrated Density – (Area of selected cell x Mean fluorescence of background readings).

### Predicted structural models of wild-type HNF1A and HNF1A mutations present in HNF1A-MODY patients (MP_1 and MP_2)

The *Homo sapiens* HNF1A CCDS 9209.1 sequence was utilized for structural predictions, which were made using Alphafold3.0 [26]. All figures were prepared with ChimeraX software [27].

### Statistical analysis

Unless indicated otherwise, data were shown as mean ± SEM of at least three independent differentiations (*N* = 3). For statistical analysis, unpaired Student’s t-tests were used to compare two groups, while one-way ANOVA or Kruskal-Wallis tests followed by Dunnett’s multiple comparisons post hoc tests were applied for multiple group comparisons. The specific statistical test used for each analysis is indicated in the corresponding figure legend. All statistical analyses were performed using GraphPad Prism software (GraphPad Software Inc., San Diego, CA, USA).

## Results

### *HNF1A* mutations trigger pronounced changes in the ECs transcriptome

**Fig. 1:**
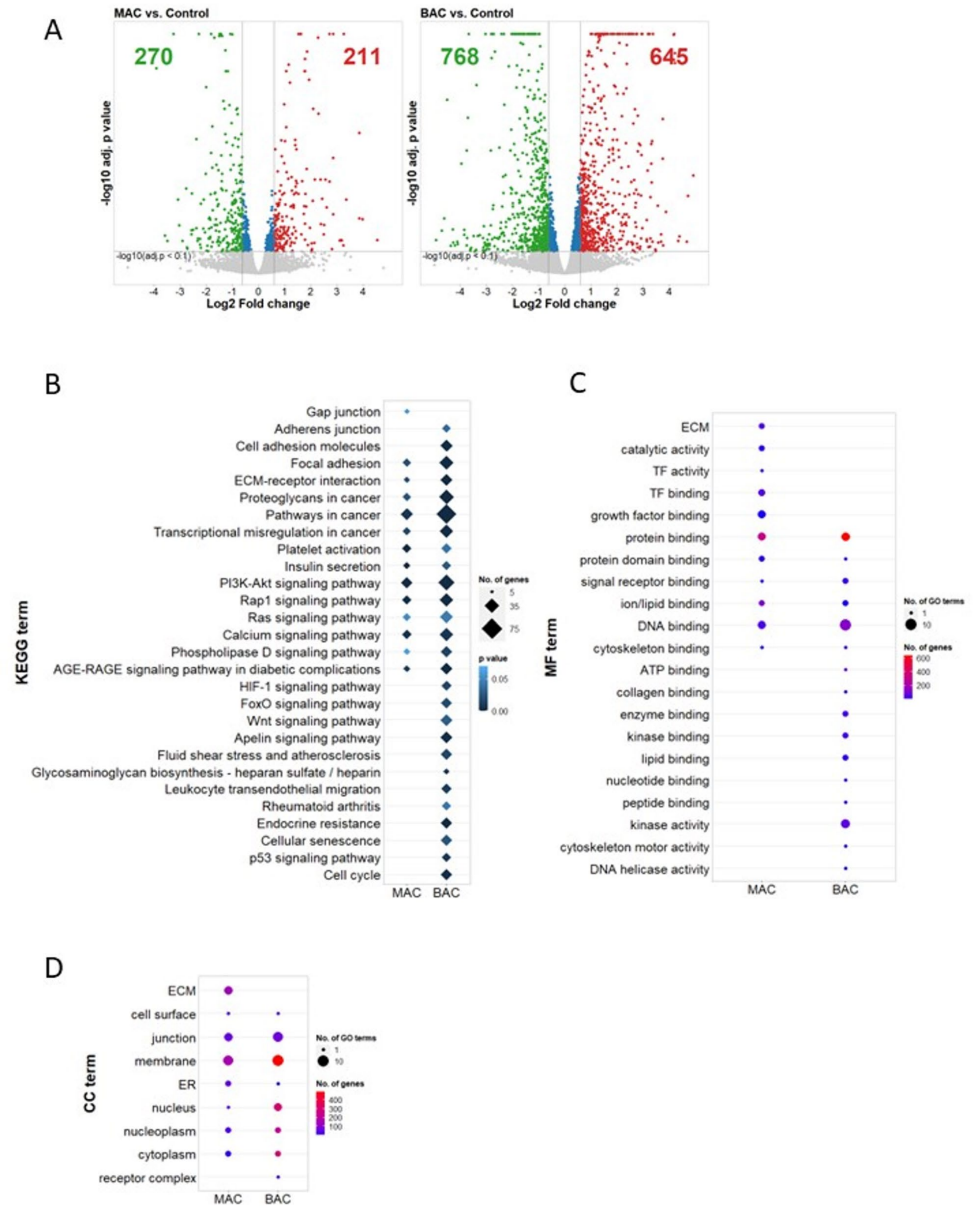
Global transcriptome analysis of the control-derived isogenic hiPSC-ECs lines (control, MAC, and BAC). **A** Volcano plots of differentially expressed genes (DEGs) in comparison to the isogenic control line. **B-D** The most representative and significant KEGG pathways, molecular function, and cellular localization of DEGs in both MAC and BAC in comparison to the isogenic control line

The detailed mechanisms of how *HNF1A* mutations affect endothelial cells (ECs) signalling and how these are linked to microvascular complications in HNF1A-MODY patients are still unknown. Therefore, to determine which molecular pathways are altered and may contribute to the dysfunctional phenotype, we performed a global transcriptome analysis using the control-derived isogenic set of hiPSC-ECs lines. Within this set, there were three cell lines: a control hiPSCs, *HNF1A* monoallelic mutation (MAC) or *HNF1A* biallelic mutation (BAC). The results showed that the biallelic mutations in *HNF1A* (BAC) had a much more pronounced impact on the ECs transcriptome. While 481 differentially expressed genes (DEGs) were detected in the MAC line, 1413 DEGs were seen in BAC in comparison to the control cells (Fig. 1A, Additional file 2: Table S1). Notably, in both mutated cell lines, the DEGs belonged to the same signalling pathways, but the effect was much more evident in BAC. The gene enrichment analysis in both mutated lines pointed to changes in genes that are involved in several pathways including focal adhesion, PI3K-Akt, Rap1, Ras, and calcium-dependent pathways. Moreover, in BAC hiPSC-ECs, there was an enrichment of genes related to cellular senescence, the p53 signalling pathway, and the cell cycle (Fig. 1B). Within the cellular compartment of the DEGs, it was noticeable that most changes are related to the cell membrane, junction or cell surface, whereas within the molecular function of DEGs, changes are mainly involved in the DNA binding, protein and ion/lipid binding (Fig. 1C, D).

**Fig. 2:**
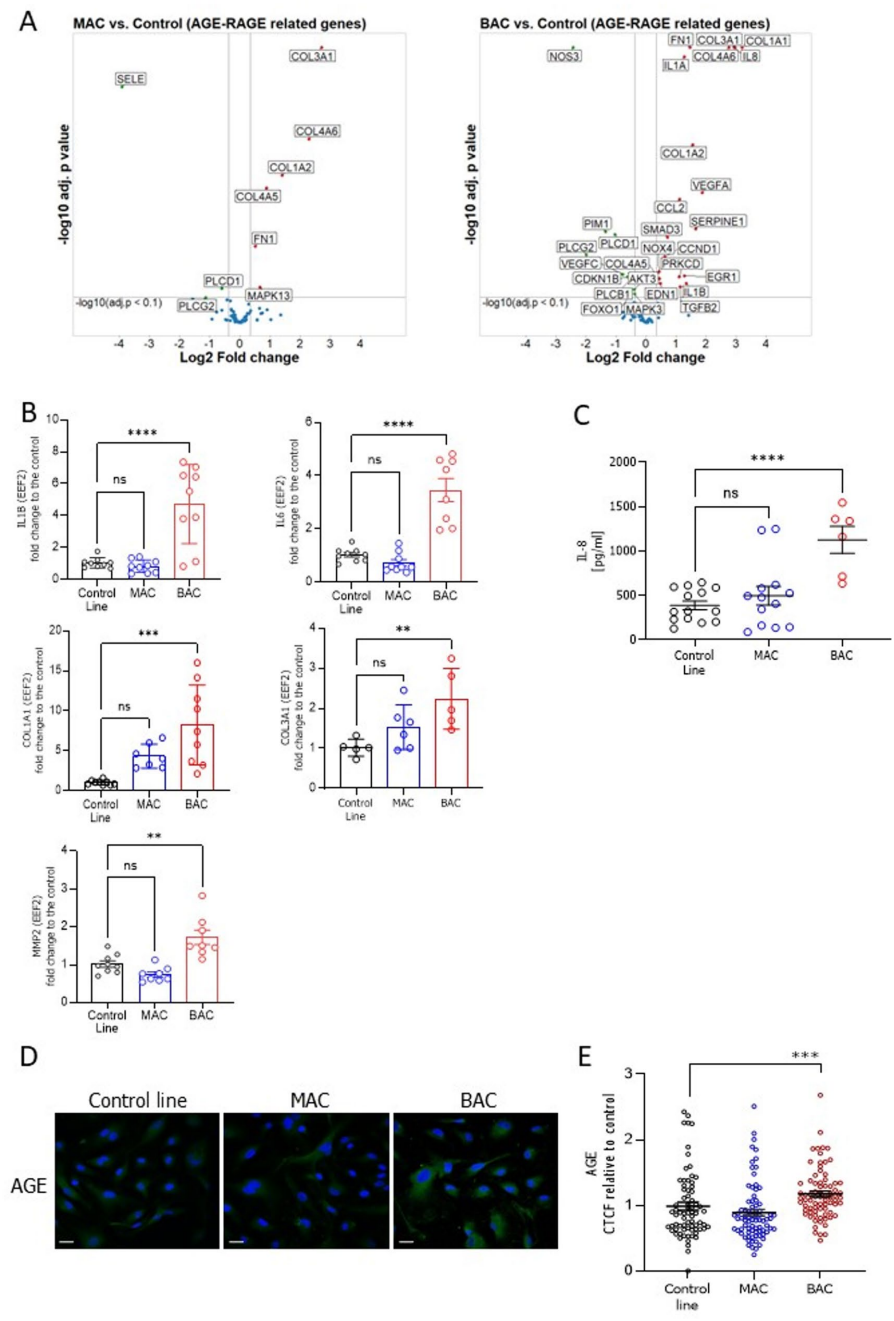
Activation of the AGE (advanced glycation products)-related, proinflammatory, profibrotic genes, and the presence of AGE in isogenic control-derived hiPSC-ECs lines (control, MAC, and BAC). **A** Volcano plots of DEGs related to AGE-RAGE pathway in MAC and BAC versus isogenic control line, results from the global transcriptome analysis. **B** Expression level of proinflammatory cytokines IL-1B, and IL-6 as well as collagen type I and collagen type III and metalloproteinase MMP2 in the isogenic set of lines. qPCR data presented as a fold change compared to the isogenic control line (*N* = 4). **C** IL-8 production measured in culture media using ELISA (*N* = 3). **D** Immunofluorescence detection of AGE (green), nuclei were visualized by DAPI (blue); scale bar 100 μm. **E** Quantitative analysis of AGE expression in the isogenic hiPSC-ECs lines based on corrected total cell fluorescence (CTCF) (*N* = 3). At least 70 cells/line were analysed. Kruskal-Wallis test, the statistically significant differences are presented as: *: *p*-value < 0.05; **: *p*-value < 0.01; ***: *p*-value < 0.001

Among the various dysregulated molecular pathways identified, a significant portion was linked to diabetes-related processes. These include key signalling pathways such as PI3K-Akt, p53, phospholipase D, and AGE-RAGE (Fig. 1B). Notably, changes in the AGE-RAGE signalling pathway could be directly related to diabetic complications (Fig. 1B). The advanced glycation end products (AGE) interact with the RAGE receptor and trigger the downstream signalling involved in the progression of atherosclerosis. At a molecular level, it promotes oxidative stress and inflammation, which is associated with the upregulation of plasminogen activator inhibitor-1 (PAI-1, *SERPINE*) and monocyte chemoattractant protein-1 (MCP-1, *CCL2*) [28]. Accordingly, in the global transcriptome analysis of the control-derived isogenic cells with mutations in *HNF1A*, we noted an increased expression of many inflammatory and profibrotic genes (Fig. 2A). The proinflammatory state was confirmed by the upregulation of cytokines such as interleukin (IL)-1B, IL-6, and IL-8 (Fig. 2B, C). As AGEs have been demonstrated to increase the expression of multiple types of collagen and generate extracellular matrix (ECM) crosslinking [29], we observed the increased expression of several collagen subsets in both MAC and BAC mutated lines (Fig. 2B). As expected, there was an increased AGE expression in BAC compared to both control and MAC lines, as shown by immunofluorescent staining (Fig. 2D, E).

**Fig. 3:**
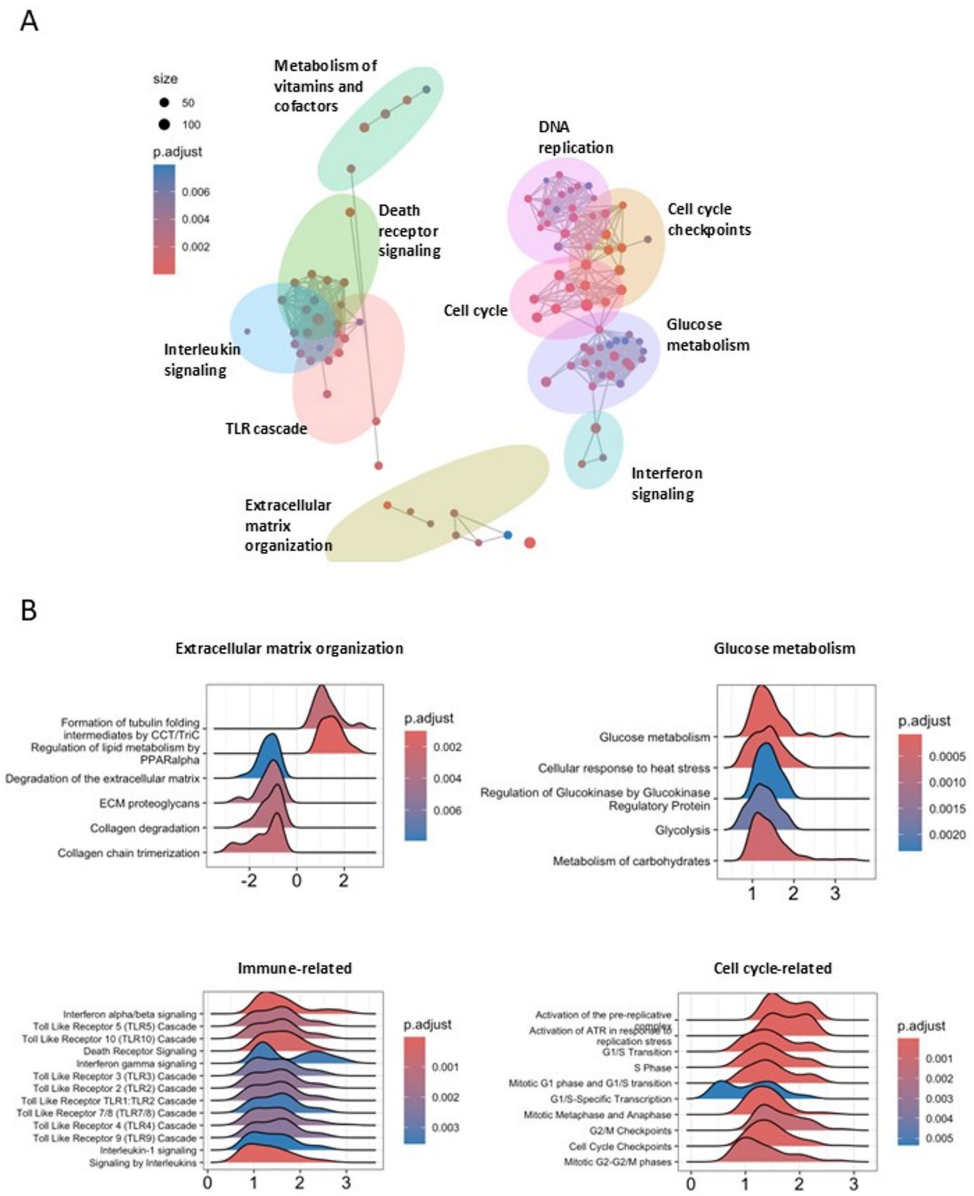
RNA-seq analysis of the patient-derived isogenic hiPSC-ECs lines (MP_1 line, corrected clone 1, and corrected clone 2). **A** Enrichment map of 100 most significantly enriched Reactome pathways clustered based on geneoverlap (coloured clouds). The size of the dots within the clouds represents the size of the set, and the colour of the dot shows the adjusted *p*-value. **B** Ridgeplots with representative pathways from four chosen classes. The x-axis represents gene log fold change divided by standard error (LFC/SE) or the Wald statistic used to rank the genes

To validate global transcriptomic alterations identified in the control-derived isogenic set, we performed RNA-seq analysis on hiPSC-ECs from the patient-derived isogenic set of lines. We first selected the 100 most enriched pathways identified by gene set enrichment analysis (GSEA) using the Reactome database [30], with genes ranked by Wald statistics. These pathways were clustered based on gene overlap using emapplot, revealing major functional modules including cell cycle, ECM organization, glucose metabolism, interleukin signalling, Toll-like receptor (TLR) signalling, and death receptor signalling (Fig. 3A; Additional file 3: Table S2). These results are consistent with those obtained from the control-derived isogenic set (Fig. 1B). To illustrate expression patterns within these functional modules, we generated ridgeplots for representative pathways from each cluster (Fig. 3B). Ridgeplots display the distribution of Wald statistics for core-enriched genes within each gene set. Pathways associated with ECM organization were predominantly downregulated, whereas immune-related pathways, cell cycle, and glucose metabolism showed overall upregulation.

### *HNF1A* mutations lead to reduced glycocalyx layer in hiPSC-ECs

**Fig. 4:**
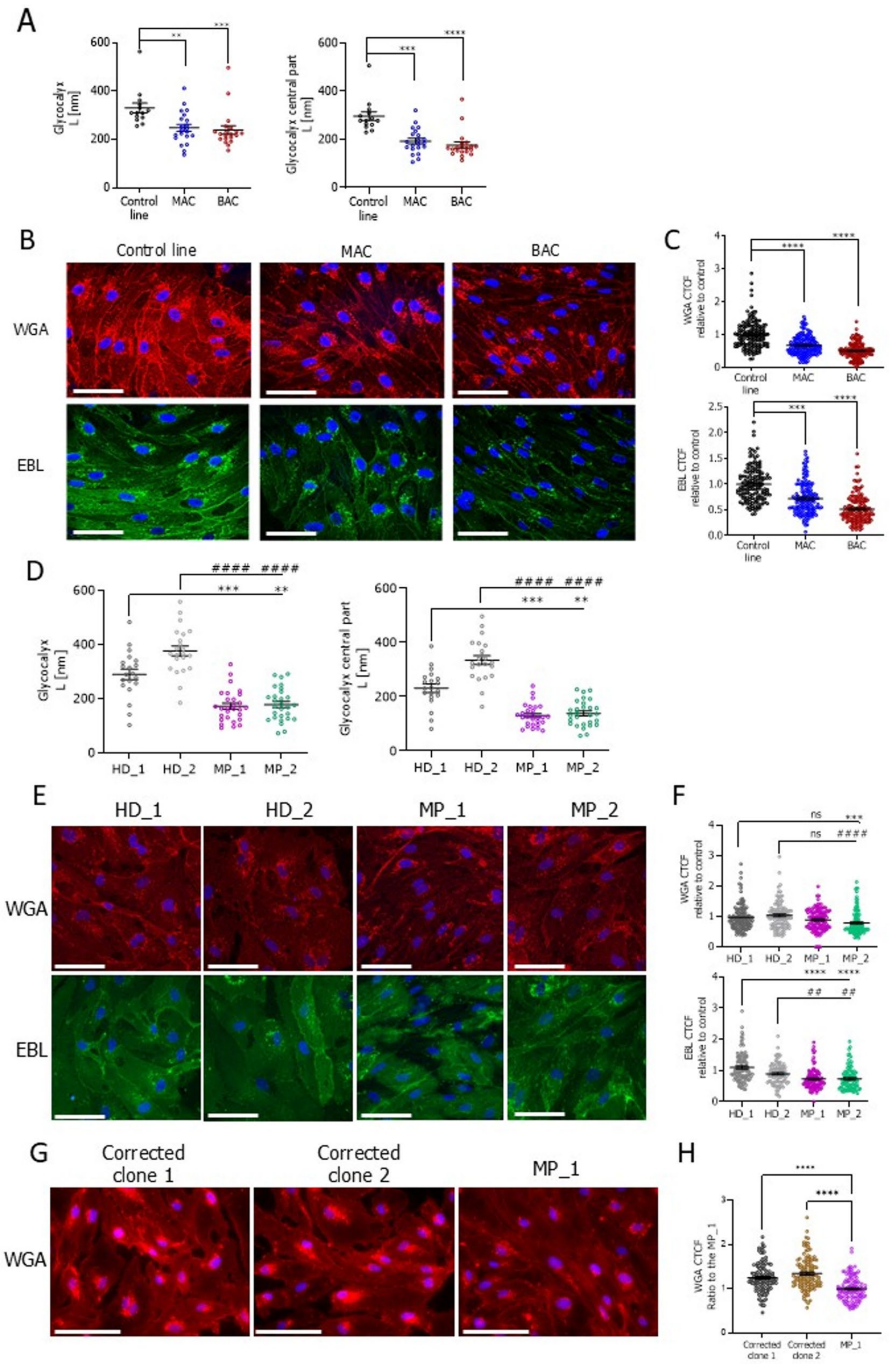
Analysis of the glycocalyx layer of the isogenic control-derived (control, MAC, and BAC), patient (HD_1, HD_2, MP_1, MP_2), and the isogenic patient-derived (Corrected clone 1, Corrected clone 2, MP_1) HNF1A-MODY sets of hiPSC-EC lines. **A** Glycocalyx length in the control isogenic set of hiPSC-ECs lines assessed with AFM. Evaluation of the general glycocalyx length and the length in the central part of the cell (*N* = 3). **B** Immunofluorescence detection of the glycocalyx components in the control isogenic hiPSC-ECs lines using lectin against heparan sulfate (WGA – red) and sialic acid (EBL – green); scale bar 100 μm. **C** Quantitative analysis of glycocalyx’s components in the control isogenic hiPSC-ECs lines based on the calculation of the corrected total cell fluorescence (CTCF) for WGA and EBL (*N* = 3). At least 70 cells/line were analysed. **D** Glycocalyx length of the patient set of hiPSC-ECs lines assessed by AFM. Evaluation of the general glycocalyx length and the length in the central part of the cell (*N* = 3). **E** Immunofluorescence detection of the glycocalyx components in the patient hiPSC-EC lines using lectin against heparan sulfate (WGA – red) and sialic acid (EBL – green); scale bar 100 μm. **F** Quantitative analysis of glycocalyx’s components in the diseased hiPSC-ECs lines based on the calculation of the corrected total cell fluorescence (CTCF) for WGA and EBL (*N* = 3). At least 70 cells/line were analysed. **G** Immunofluorescence detection of the glycocalyx components in the isogenic patient-derived hiPSC-EC lines using lectin against heparan sulfate (WGA – red); scale bar 100 μm. **H** Quantitative analysis of glycocalyx’s components in the isogenic patient-derived hiPSC-ECs based on the calculation of the corrected total cell fluorescence (CTCF). At least 70 cells/line were analysed. Data presented as mean ± SEM; Kruskal-Wallis test, the statistically significant differences are presented as: ** or ##: *p*-value < 0.01; ***: *p*-value < 0.001; **** or ####: *p*-value < 0.0001. In figures depicting the results for the patient set, asterisks (*) were used to compare the HD_1 hiPSC-ECs with patient-derived hiPSC-ECs lines (MP_1 and MP_2) and hash marks (#) to compare the HD_2 hiPSC-ECs with patient-derived hiPSC-ECs lines (MP_1 and MP_2)

The increase in AGE, detected with the control-derived isogenic set, can trigger a pro-inflammatory state of the ECs, which may further disturb the endothelial glycocalyx. Moreover, RNA-seq analysis of the patient-derived isogenic set reveals downregulation of the ECM proteoglycans pathway, further suggesting alterations in the glycocalyx layer of *HNF1A*-mutated cells. This brush-like layer of membrane-bound carbohydrate-rich molecules has a significant role in endothelial homeostasis and is frequently affected in different disease states. The reduction of glycocalyx in diabetes can be detected before the clinical manifestation caused by increased vascular permeability and subsequent retinopathy or nephropathy [31]. In hiPSC-ECs from the control-derived isogenic set, there was a clear shortening of the glycocalyx layer when *HNF1A* was mutated in one or both alleles. This was shown by atomic force microscope (AFM) analysis (Fig. 4A) as well as by staining of sialic acid and heparan sulfate decoration on hiPSC-ECs (Fig. 4B, C). Similarly, in the HNF1A-MODY patient-derived hiPSC-ECs, a clear shortening and reduced decoration of the glycocalyx layer was observed in comparison to the healthy donor-derived counterparts (Fig. 4D-F). Importantly, following CRISPR/Cas9-mediated correction of the *HNF1A* mutation in the MP_1 line, we observed restoration of glycocalyx layer decoration (Fig. 4G-H).

### *HNF1A* mutations trigger pronounced changes in the ECs proteome

**Fig. 5:**
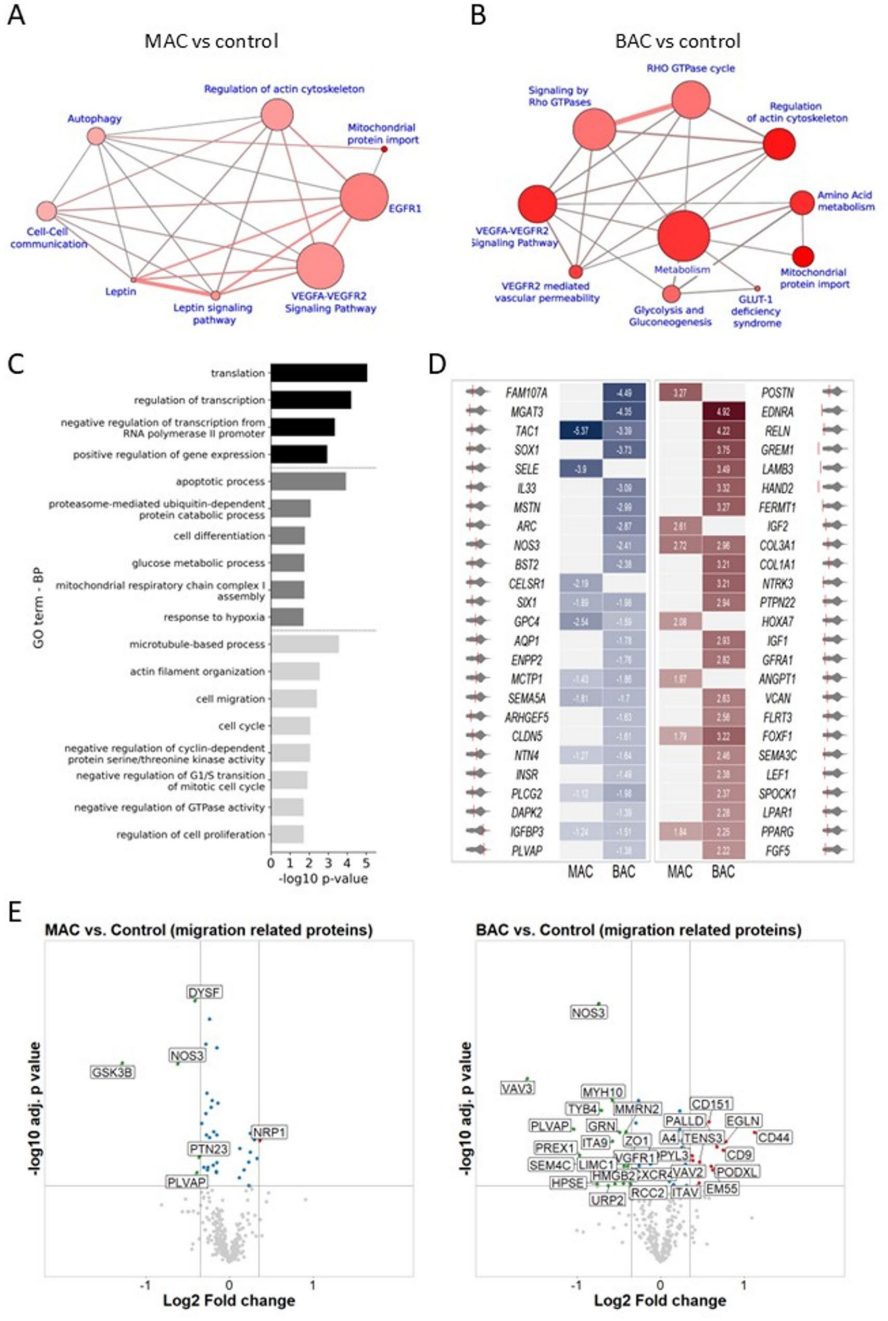
Proteomic analysis of the control-derived isogenic hiPSC-ECs lines (control, MAC, and BAC). **A-B** Enrichment analysis of DEPs in the pathway–based sets of MAC and BAC created with ConsensuspathDB. Each node represents a separate concept for which size and *p*-value are encoded as node size and node colour, respectively. The edge colour encodes the number of shared members. **C** Enrichment analysis of 328 genes that were differentially expressed in BAC and were on the list of HNF1A putative targets, as identified by ChIP-seq analysis in Chan et al., 2023. The analysis of the biological processes was performed with DAVID software. **D** Top 25 up and down-regulated genes, related to cell migration in both MAC and BAC compared to the isogenic control line. **E** Volcano plots of migration-related DEP in MAC and BAC compared to the isogenic control line

Taking a step further, we wanted to see how the *HNF1A* mutation-related transcriptomic changes translate to the proteome. The global proteome analysis revealed 86 and 116 differentially expressed proteins (DEPs) in MAC and BAC, respectively (Additional file 1: Fig. S9). The over-representation analysis of these DEPs showed pathways related to the actin cytoskeleton, Rho GTPases, VEGFR2 signalling, and mitochondrial protein import (Fig. 5A, B). The gene ontology (GO) terms related to cell adhesion, cell-substrate junction, ion binding, and mitochondrial protein complex (Additional file 1: Fig. S10A, B). Additionally, in hiPSC-ECs with the biallelic mutations, DEPs were also clustered in pathways related to cell metabolism and glycolysis (Fig. 5B). These results are in line with the changes observed in the transcriptome data. Moreover, comparing the 2800 HNF1A putative targets, identified by HNF1A ChIP-seq analysis in kidney organoids [32] and the DEGs in BAC, more than 300 common genes were revealed (hypergeometric probability test 0.00104, Additional file 4: Table S3). The downstream analysis of these common genes revealed that they are involved in different biological processes like transcriptional activity, apoptosis, glucose metabolism, and cell differentiation (Fig. 5C). Interestingly, many processes could be related to the cell cytoskeleton like migration, proliferation, cell cycle, and microtubule-based processes.

Both global analyses of the transcriptome and the proteome of hiPSC-ECs, from the control-derived isogenic set of lines, with mutations in the *HNF1A* showed changes related to the cytoskeleton. It is known that the mechanism of cell migration involves the coordinated dynamics of the actin cytoskeleton, cell–cell communication as well as contact of the cell with the ECM [33]. Moreover, the migratory capacity is one of the pivotal features of endothelial function implicated in vascular regeneration [34]. When looking for DEGs involved in the process of cell migration, there were 74 genes in MAC and 204 in BAC (Additional file 5: Table S4). Each DEG was ranked based on the fold change in MAC and BAC, and then the averaged top 25 up-regulated and down-regulated genes were depicted in Fig. 5D. There was an up-regulation in collagen 1 and 3 genes, which could alter the ECM environment of the mutated cells and thus affect the cell migration. Additionally, in both MAC and BAC there was an upregulation in *PPARG* (peroxisome proliferator-activated receptor gamma), which was previously shown to have a role in the ECs migration through induction of the expression of *Sema3g* (semaphorin 3G) [35]. When looking at the proteomic results, only a few DEPs related to cell migration were significantly affected in MAC, whereas more than twenty were found in BAC (Fig. 5E).

### Mutations in the *HNF1A* increase the ECs migratory capacity and this effect could be mimicked by silencing of HNF1A in primary ECs

**Fig. 6:**
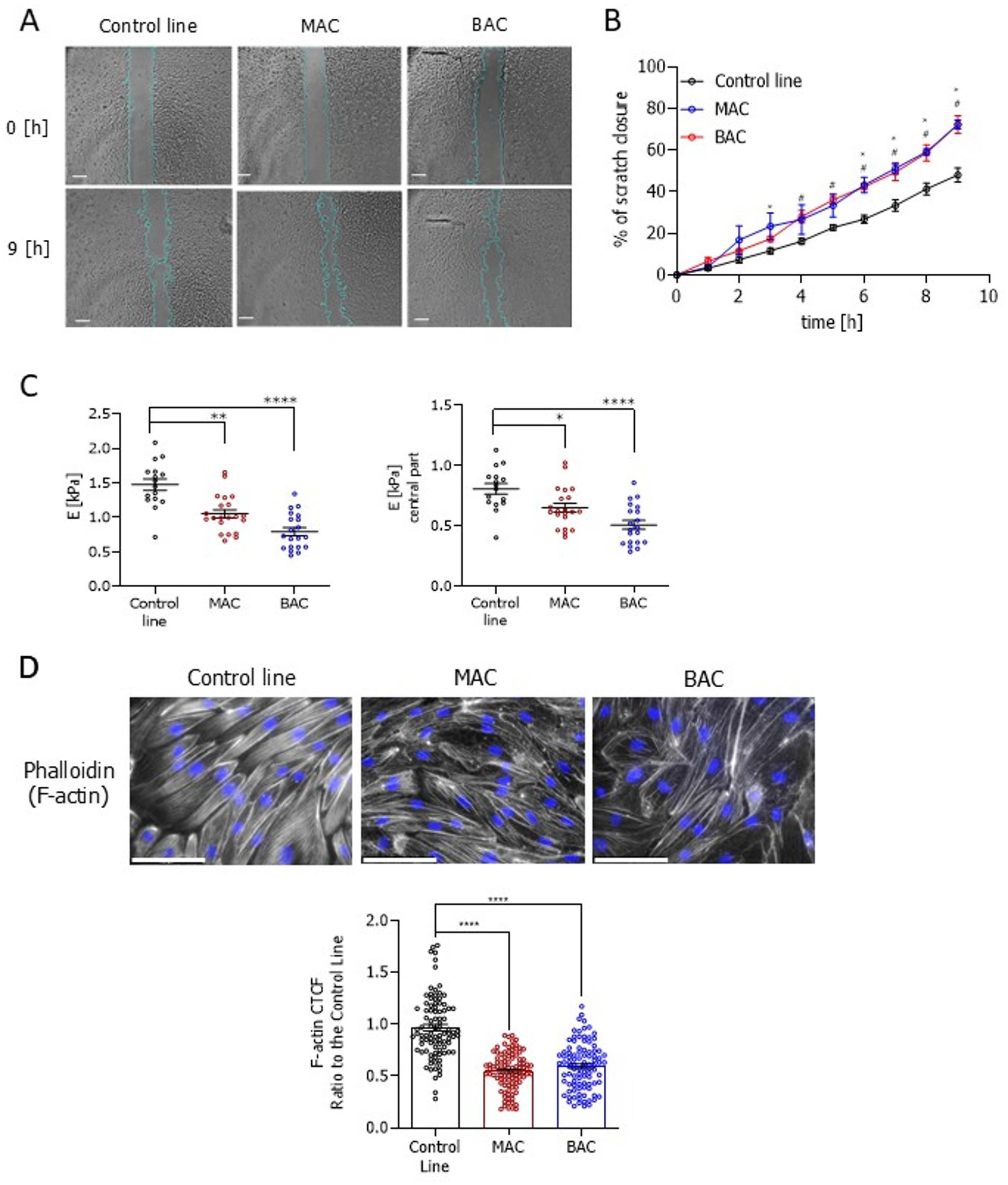
Migratory potential and stiffness of the control-derived isogenic (control, MAC, and BAC) set of hiPSC-ECs lines. **A** Time-lapse images (time points: 0 h and 9 h) of scratch closure of the isogenic hiPSC-ECs lines; scale bar 500 μm. **B** Quantitative analysis of the migratory potential of isogenic hiPSC-ECs lines presented as a percentage of scratch closure (*N* = 5). **C** Elastic modulus of the isogenic hiPSC-ECs, measured by AFM, presented for the whole area and for the central part of a cell (*N* = 3). At least 16 cells/line were measured. **D** Immunofluorescence pictures and quantification based on the calculation of the corrected total cell fluorescence (CTCF) of actin cytoskeleton structure in the isogenic set of lines: actin cytoskeleton (phalloidin – white), nuclei (DAPI – blue); scale bar 100 μm. At least 70 cells/line were analysed. In Fig. **B** asterisks (*) were used to compare MAC with the control line and hash marks (#) to compare BAC with the control line. Data presented as mean ± SEM; Kruskal-Wallis test, the statistically significant differences were presented as: *: *p*-value < 0.05; **: *p*-value < 0.01; ***: *p*-value < 0.001. ****: *p*-value < 0.0001

To assess how cytoskeleton-related changes observed in transcriptomic and proteomic analyses affect cell phenotype, a migration assay was conducted. We observed that both mutated lines from the control-derived isogenic set had higher migratory potential in comparison to the isogenic control counterpart (Fig. 6A, B). Since many of the genetic and proteomic changes are related to the cytoskeleton, we tested for cytoskeleton modification in MAC and BAC cells by measuring the cellular stiffness. To achieve this, we performed AFM analysis, which revealed that the mutations in *HNF1A* result in decreased stiffness in comparison to the control cells of the control-derived isogenic set (Fig. 6C). This was true both for the central region of the cell and for the whole cell, confirming the specificity of the measurement. In accordance, actin stress fibres were significantly less abundant and organized in MAC and BAC hiPSC-ECs (Fig. 6D).

**Fig. 7:**
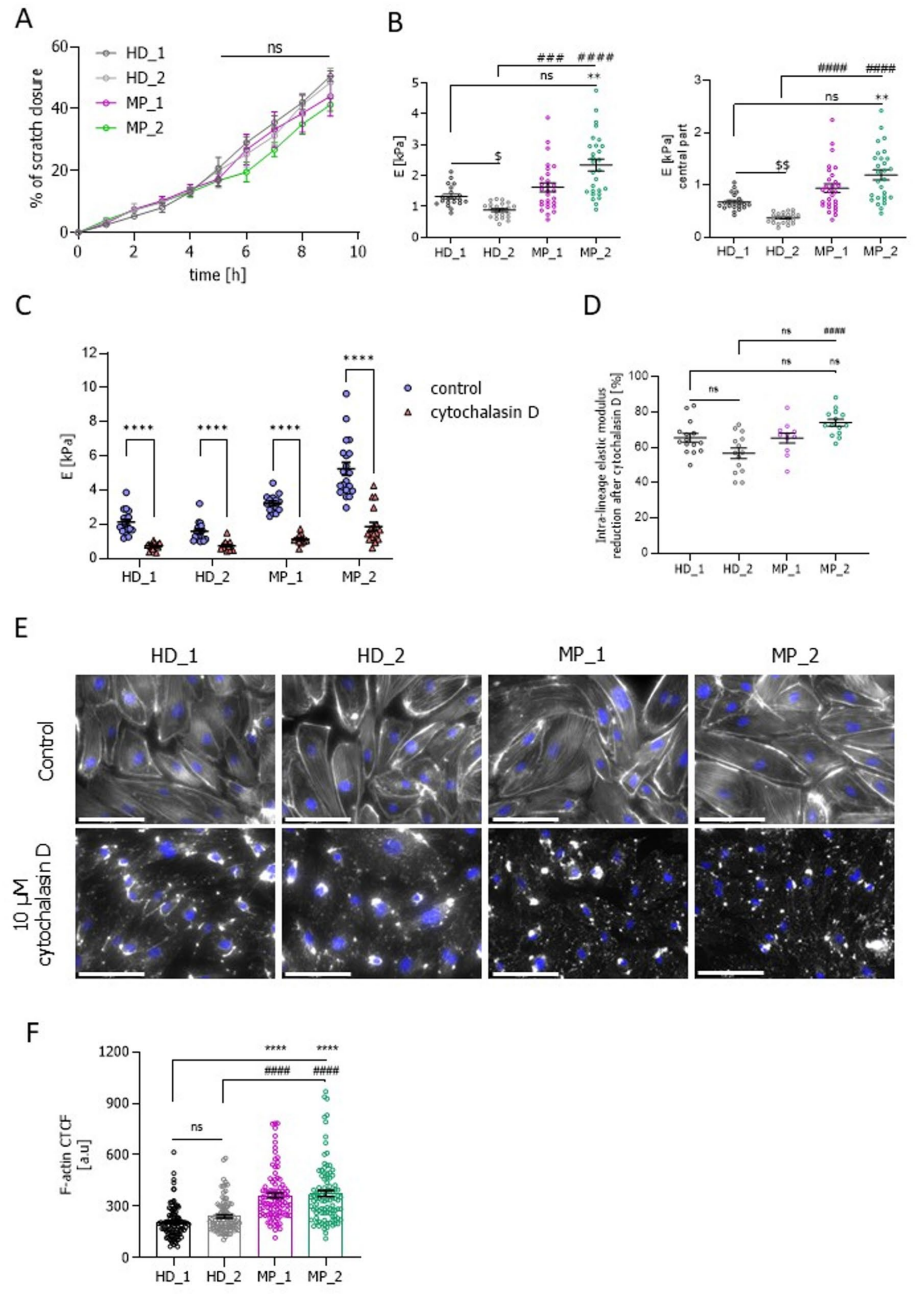
Migratory potential and stiffness of the patient (HD_1, HD_2, MP_1, and MP_2) set of hiPSC-ECs lines. **A** Quantitative analysis of the migratory potential of the patient hiPSC-ECs lines, presented as a percentage of scratch closure (*N* = 4). **B** Elastic modulus of the patient hiPSC-ECs lines, measured by AFM, presented for the whole area and for the central part of a cell (*N* = 3). At least 20 cells/line were measured. **C** Elastic modulus of the patient hiPSC-ECs set of lines, measured by AFM before (control) and after treatment with cytochalasin D. At least 16 cells/line were measured. **D** Intra-lineage reduction of the elastic modulus after cytochalasin D (*N* = 3). **E** Immunofluorescence pictures of actin cytoskeleton structure in control conditions and after cytochalasin D: actin cytoskeleton (phalloidin – white), nuclei (DAPI – blue); scale bar 100 μm. **F** Quantification of F-acting in the control conditions (Fig. 7E upper panel), based on the calculation of the corrected total cell fluorescence (CTCF). At least 70 cells/line were analysed. Data presented as mean ± SEM; unpaired t-test (C) or one-way Anova tests, the statistically significant differences were presented as: * or # or $:*p*-value < 0.05; ** or $$: *p*-value < 0.01; *** or ###: *p*-value < 0.001. **** or ####: *p*-value < 0.0001. In Figures **B**, **D** and **F** depicting the results for the patient set of lines, asterisks (*) were used to compare the HD_1 hiPSC-ECs with HNF1A-MODY-derived hiPSC-EC lines, whereas hash marks (#) were used to compare the HD_2 hiPSC-ECs with MP_1 and MP_2, while the dollar mark ($) was used to compare the control line HD_2 to HD_1

To check if this same phenotype could be found in cells with other *HNF1A* mutations, we analysed the migratory capacity and cellular stiffness in hiPSC-ECs derived from HNF1A-MODY patients in comparison to hiPSC-ECs of healthy donors. Contrary to the control-derived isogenic set of hiPSCs lines, there was no difference in the migratory capacity of these cells in comparison to the healthy donors set of hiPSC-ECs (Fig. 7A, Additional file 1: Fig. S11). However, we observed increased stiffness (Fig. 7B) measured with AFM and more abundant presence of actin stress fibres in cells derived from HNF1A-MODY patients in comparison to the healthy donor counterparts (Fig. 7E upper panel and 7F). Interestingly, there was also a significant difference between the two healthy controls, with HD_2 being less stiff than HD_1, showing that the genetic background and patient variation could have an impact on the analysed parameters.

To investigate whether the increased presence of actin stress fibres contributed to the elevated stiffness in the patient-specific lines, we conducted additional AFM measurements before and after treating the HNF1A-MODY hiPSC-EC lines with cytochalasin D. Cytochalasin D disrupts actin dynamics by inducing actin depolymerization and inhibiting its polymerization [36]. The reduction of stiffness after treatment with cytochalasin D was substantial across all lines (Fig. 7C). HD_1 and the two patient-derived lines showed a similar drop in cell stiffness after treatment with cytochalasin D. In HD_2, where the initial stiffness was less pronounced, the effect of cytochalasin D was less prominent (Fig. 7D). This proves that the observed cell stiffness can be correlated with the presence of the actin stress fibres in the cells. The effect of cytochalasin D on the actin architecture was shown in Fig. 7E (lower panel).

**Fig. 8:**
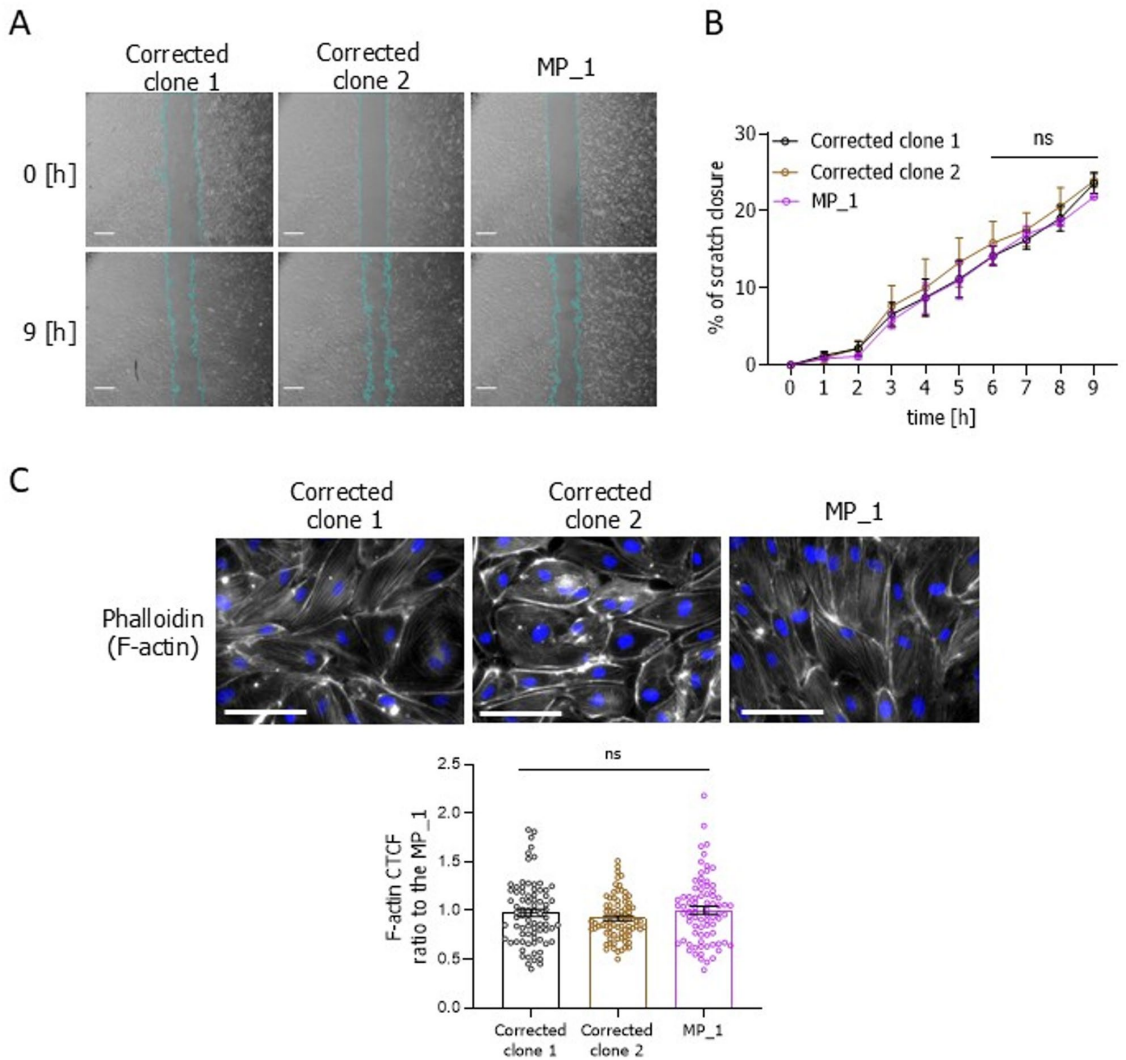
Migratory potential and actin stress fibres quantification in the isogenic patient-derived (Corrected clone 1, corrected clone 2, MP_1) set of hiPSC-ECs lines. **A** Time-lapse images (time points: 0 h and 9 h) of scratch closure of the isogenic patient-derived hiPSC-ECs; scale bar 500 μm. **B** Quantitative analysis of the migratory potential of the isogenic patient-derived hiPSC-ECs lines, presented as a percentage of scratch closure (*N* = 3). **C** Immunofluorescence pictures and quantification based on the calculation of the corrected total cell fluorescence (CTCF) of actin cytoskeleton structure in the isogenic set of lines: actin cytoskeleton (phalloidin – white), nuclei (DAPI – blue); scale bar 100 μm. At least 70 cells/line were analysed. Data presented as mean ± SEM; Kruskal-Wallis test

**Fig. 9:**
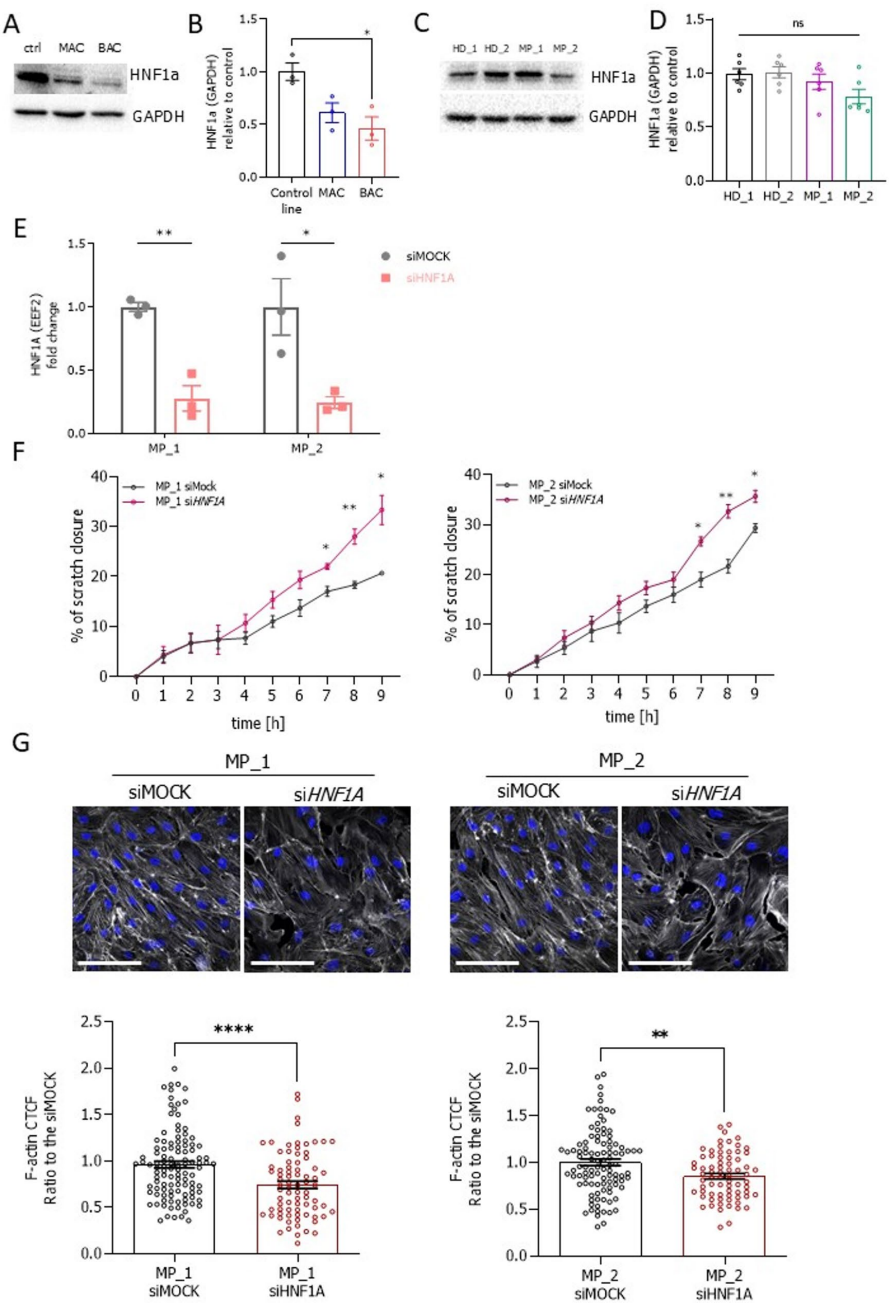
Expression of HNF1A in the control-derived isogenic (control, MAC, and BAC) and patient (HD_1, HD_2, MP_1, MP_2) HNF1A-MODY sets of hiPSC-ECs lines and migratory potential of HNF1A-MODY-derived hiPSC-EC lines (MP_1, and MP_2) with reduced expression of *HNF1A*. **A-D** Western blot analysis of HNF1A level in the isogenic (*N* = 3) and patient hiPSC-ECs lines (*N* = 6), GAPDH was used as a loading control. **E** Silencing efficiency of *HNF1A* mRNA expression in patient-derived hiPSC-ECs lines MP_1 and MP_2 72 h-post transfection (*N* = 3). **F** Time-lapse images (time points: 0 h and 9 h) of scratch closure of MP_1 and MP_2 hiPSC-ECs lines transfected with siMOCK or siHNF1A and quantitative analysis, presented as a percentage of scratch closure; scale bar 500 μm (*N* = 3). **G** Immunofluorescence pictures and quantification of the actin cytoskeleton architecture in MP_1 and MP_2 lines with a silenced expression of *HNF1A*: actin cytoskeleton (phalloidin – white), nuclei (DAPI – blue); scale bar 100 μm. At least 70 cells/line were analysed. Data presented as mean ± SEM; Kruskal-Wallis test or unpaired t-test, the statistically significant differences were presented as: *: *p*-value < 0.05; **: *p*-value < 0.01; ****: *p*-value < 0.0001

To evaluate whether the correction of *HNF1A* mutation in the MP_1 line would affect endothelial stiffness and cell migration, we stained for phalloidin and performed migration assays (Fig. 8A, B). The corrected clones had similar actin stress fibres as compared to the original MP_1 line (Fig. 8C), meaning that the restoration of the mutated allele did not affect the cellular stiffness. Expectedly, the migration efficiency of the corrected clones did not differ from the original MP_1 line (Fig. 8A, B). One possible reason for the discrepancy in migration and cellular stiffness between the control-derived isogenic set of lines and the patient-derived hiPSC-ECs could be the genetic background of the diabetes-diagnosed HNF1A-MODY patients. Another possible reason could be the level of HNF1A in hiPSC-ECs. Even though the HNF1A expression in ECs is quite low in comparison to the hepatocellular cell line HepG2, we were able to specifically detect this protein using Western blot analysis (Additional file 1: Fig. S12). While BAC had a significantly reduced level of HNF1A (Fig. 9A, B), it did not differ between the patient-derived and control cell lines (Fig. 9C, D). Therefore, to verify whether the presence of HNF1A could be responsible for the effect on the cell migration, we silenced *HNF1A* in patient-derived hiPSC-ECs, using siRNA. Even though the decrease of *HNF1A* on the mRNA level was only partial (Fig. 9E), such silencing was sufficient to affect the migratory capacity of these cells. As shown in Fig. 9F and Additional file 1: Fig. S12B, the decrease of *HNF1A* in patient-derived cells increased their migratory capacity as compared to mock-transfected cells, which was accompanied by a reduction in the actin stress fibres (Fig. 9G). These results suggest that the cell stiffness and the migration capacity in HNF1A-MODY patient-derived hiPSCs could be the cumulative result of diabetic epigenetic changes and/or HNF1A expression in these cells.

**Fig. 10:**
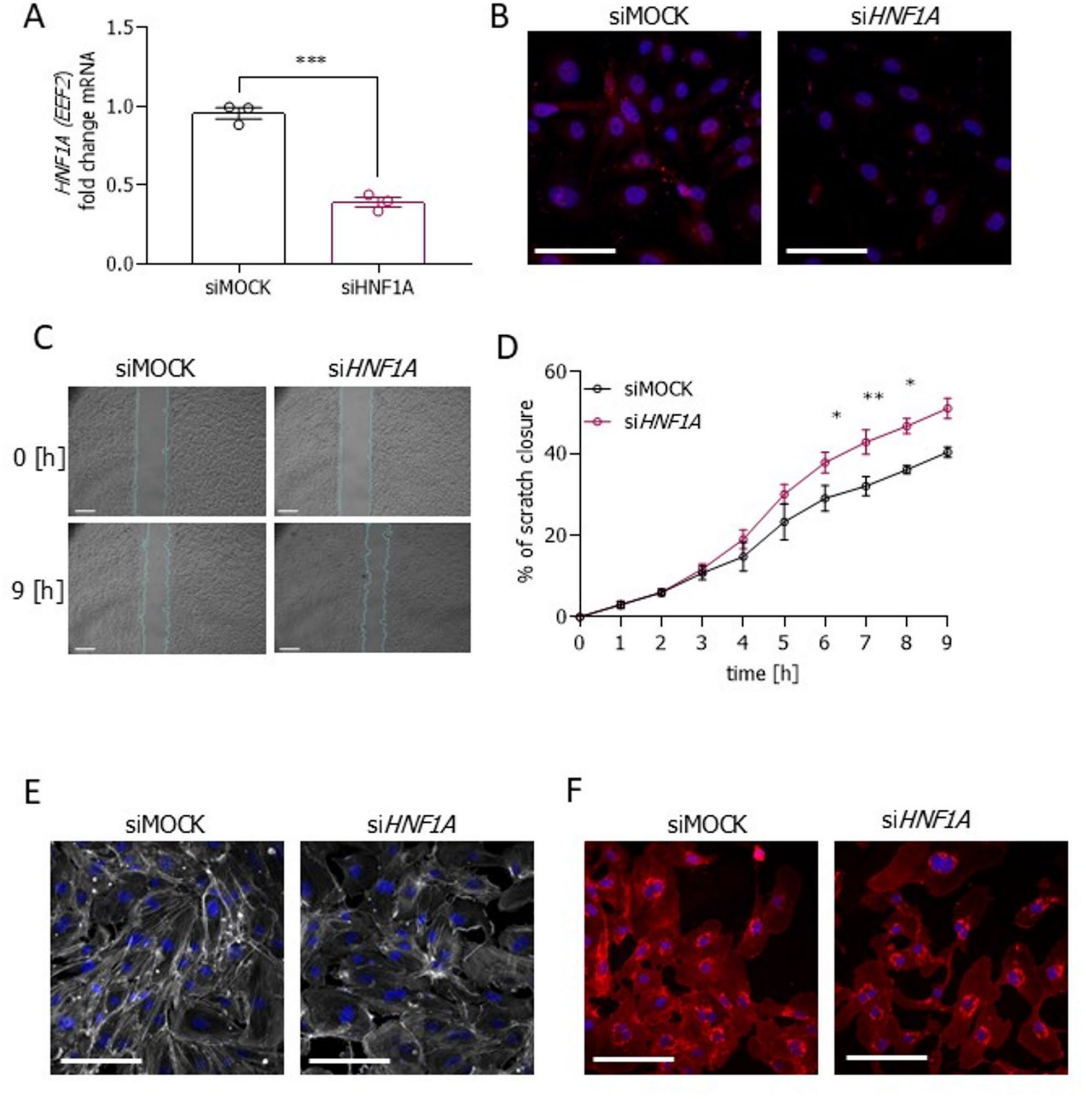
Reduced expression of HNF1A in primary ECs affects the migratory potential and glycocalyx layer. **A** mRNA level of the HNF1A gene in human aortic endothelial cells (HAECs) transfected with siMOCK or siHNF1A; data were presented as a fold change versus control (*N* = 3). **B** Immunofluorescence detection of HNF1A: HNF1A (red), nuclei (DAPI – blue); scale bar 100 μm. **C** Time-lapse images (time points: 0 h and 9 h) of scratch closure of HAECs transfected with siMOCK or siHNF1A; scale bar 500 μm. **D** Quantitative analysis of HAECs transfected with siMOCK or siHNF1A presented as a percentage of scratch closure (*N* = 3). **E** Immunofluorescence pictures of the actin cytoskeleton architecture in HAECs transfected with siMOCK or siHNF1A: actin cytoskeleton (phalloidin – white), nuclei (DAPI – blue); scale bar 100 μm. **F** Immunofluorescence detection of the glycocalyx in HAECs transfected with siMOCK or siHNF1A: heparan sulfate (WGA – red); scale bar 100 μm. Data presented as mean ± SEM, unpaired t-test, the statistically significant differences were presented as: *: *p*-value < 0.05 **: *p*-value < 0.01, ***: *p*-value < 0.001

To verify whether the effect of *HNF1A* silencing could also be observed in primary ECs, we used human aortic endothelial cells (HAECs), in which we silenced *HNF1A* using siRNA. Importantly, *HNF1A*-deficient HAECs (Fig. 10A, B) recapitulated the phenotype of hiPSC-ECs from the control-derived isogenic set of lines, showing increased migration capacity (Fig. 10C, D), decreased actin cytoskeleton polymerization (Fig. 10E), and reduced glycocalyx length (Fig. 10F). All these experiments show a clear impact of the HNF1A protein on the ECs phenotype.

### Characterization of *HNF1A* mutations in the patient-derived cell lines

The majority of functional studies on HNF1A mutations focus on the highly conserved DNA-binding domain (DBD) [17]. In our current investigation, we targeted this specific region to generate isogenic cell lines for functional analysis. Previous research has demonstrated that mutations within the DBD, such as the insertion mutation (c.422_423insT, p.Gln141HisfsX47), significantly reduce HNF1A DNA-binding activity [37]. Furthermore, data from the ClinVar database indicate that the majority of frameshift mutations in HNF1A (76 out of 83) result in pathogenic outcomes, largely due to haploinsufficiency or the production of truncated, non-functional HNF1A variants [38]. This suggests that frameshift mutations represent one of the most deleterious mutation types in HNF1A, severely disrupting its function.

Building on these findings, in this study, we utilized patient-derived cell lines harbouring mutations that disrupt the DBD (Table 1). The MP_1 cell line carries a frameshift mutation (c.235_236insG) that leads to a premature stop codon (p.Glu79GlyfsX16), resulting in the loss of the DBD and other following domains. This mutation occurs within the linker region between the dimerization domain (DD) and the DBD, a crucial juncture for HNF1A’s structural integrity. This mutation is located near another known pathogenic mutation (c.142delG, p.Glu48SerfsX155), which has been shown to severely impair HNF1A’s transcriptional activity [39].

The MP_2 cell line presents a missense mutation (c.824 A > T), resulting in a single amino acid substitution (p.Glu275Val) within the DBD. While this specific mutation has not been studied in depth, similar mutations affecting neighbouring residues (e.g., p.Arg271Gln and p.Arg272His) have been associated with significantly reduced HNF1A transcriptional activity and DNA-binding affinity [40–42].

### Prediction of the HNF1A structures in the patient-derived HNF1A-MODY lines

To investigate the structural impact of *HNF1A* mutations on protein function, we conducted in silico modelling utilizing the AlphaFold 3.0 protein structure prediction tool. Given that HNF1A acts as a transcription factor only after dimerization, and that the disease-causing mutations in patients are mainly monoallelic, we explored all possible dimer combinations between the wild-type (WT) and the mutated HNF1A variants. We analysed the structures both in the presence and absence of the palindromic HNF1A-specific DNA binding fragment. The protein core had high prediction confidence, based on the pIDDT prediction score (Additional file 1: Fig. S13).

**Fig. 11:**
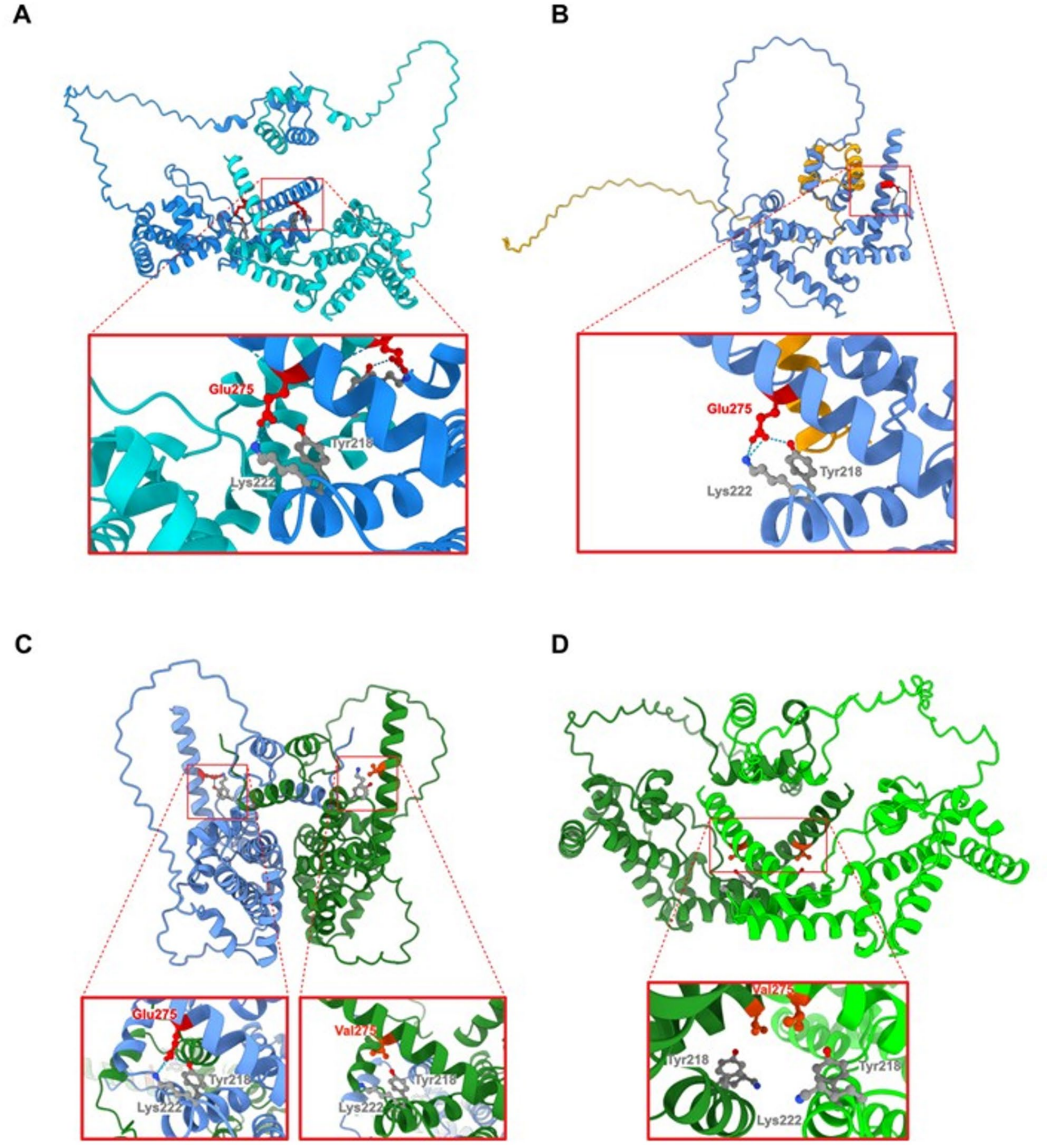
Overall, AlphaFold 3.0 predicted structures of: (**A**) HNF1A WT homodimer, (**B**) HNF1A WT-MP1 heterodimer, (**C**) HNF1A WT-MP2 heterodimer, and (**D**) HNF1A MP2-MP2 homodimer (upper panels) with magnified indicated areas (lower panels) showing local environment of the residue no. 275 together with the network of hydrogen bonds (dashed light blue lines), if present. HNF1A WT in blue, HNF1A MP1 in gold, and HNF1A MP2 in green; protein fold is shown as ribbon, and residues involved in H-bonding are shown as ball-and-sticks. Unstructured regions of the protein were hidden to enhance figure clarity

The truncation observed in the HNF1A-MP_1 variant results in the complete absence of the DNA-binding domain, including the nuclear localization signal (NLS) and the transactivation domain (Fig. 11B), in comparison to the wild-type homodimer (Fig. 11A). This truncation has significant functional implications. The absence of the NLS suggests that the HNF1A-MP_1 variant would be unable to efficiently translocate into the nucleus, thus preventing its ability to bind to DNA and exert transcriptional control. Furthermore, even when dimerized with WT-HNF1A, the truncated HNF1A-MP_1 is unlikely to contribute effectively to transcriptional activity due to the loss of the DNA-binding and transactivation domains.

The HNF1A-MP_2 variant, characterized by a single amino acid substitution of glutamic acid (Glu) to valine (Val) at position 275, resides within the DNA-binding domain. This mutation leads to a significant structural rearrangement in the local environment of the dimerization interface (Fig. 11C and D). In the WT structure, Glu275 forms stabilizing hydrogen bonds with Lys222 and Tyr218, which are crucial for maintaining the integrity of the DNA-binding domain. The substitution of Glu275 to Val in the HNF1A MP_2 variant abolishes this critical network of hydrogen bonds. This destabilization propagates through the protein, altering the structure of the dimerization domain, particularly when the HNF1A MP_2 variant forms a homodimer with itself (MP_2) or a heterodimer with WT-HNF1A (WT-MP_2) (Fig. 11C and D).

**Fig. 12:**
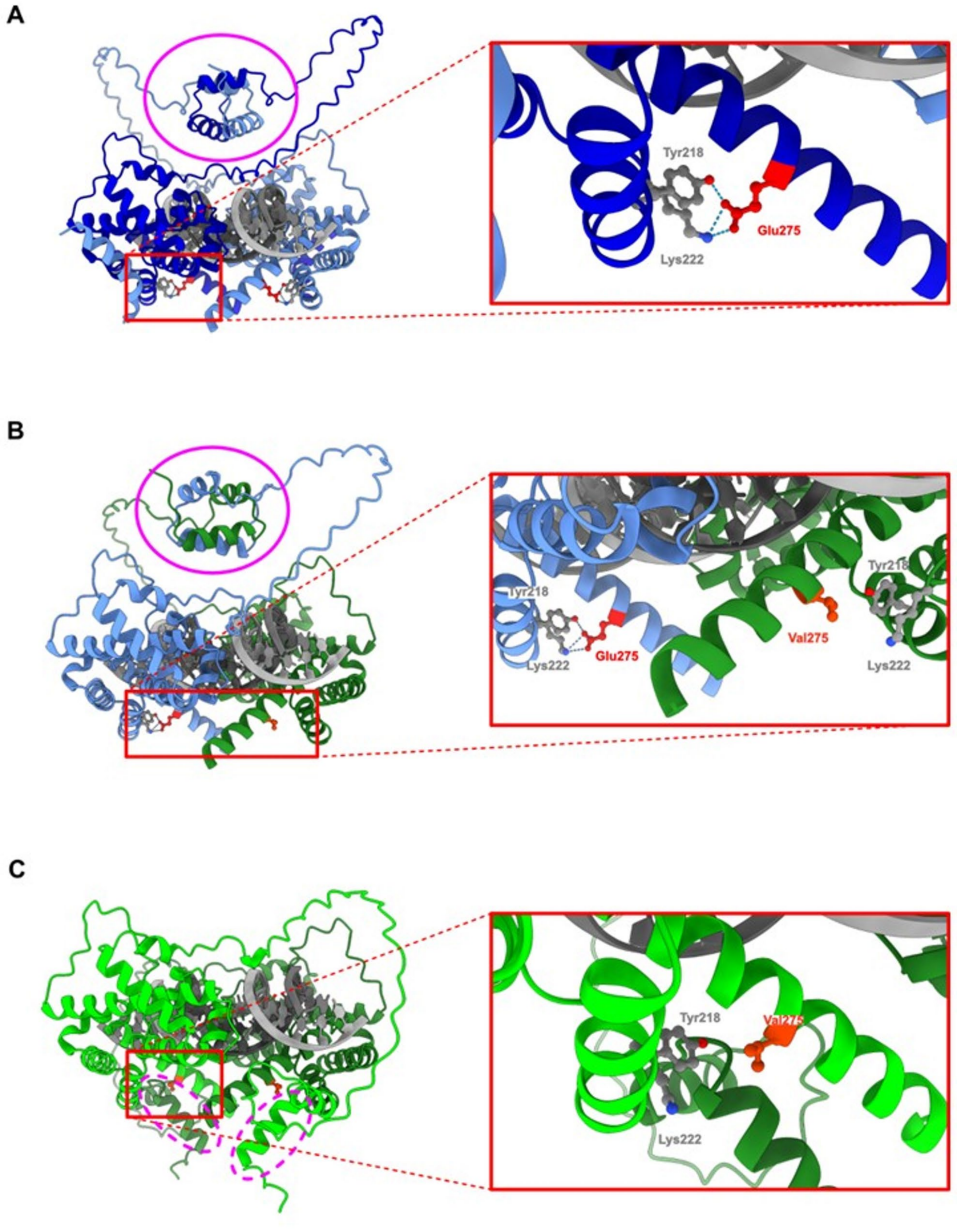
Overall, AlphaFold 3.0 predicted structures of HNF1A together with bound dsDNA fragment: (**A**) HNF1A WT dimer, (**B**) HNF1A WT-MP2 dimer (heterodimer), (**C**) HNF1A MP2-MP2 dimer (mutated homodimer), (left panels). Magnified indicated area (right panels) showing the same residue position (275) and H-bonds network as in the case of the dimers without dsDNA bound. The dimerization domain marked with a magenta ellipse appeared to be disrupted in the case of HNF1A MP2-MP2 homodimer (marked with a magenta dashed ellipse). Unstructured regions of the protein were hidden to enhance figure clarity

In the context of DNA binding, the structural changes induced by the MP_2 mutation are particularly evident as compared to the WT homodimer (Fig. 12A). When the heterodimer of HNF1A with WT and MP_2 interacts with the DNA, the overall structure remains somewhat intact, but a noticeable distortion is observed in the dimerization interface, which together with loss of hydrogen bonds could impact the stability and efficiency of DNA binding (Fig. 12B). In contrast, the homodimer formed exclusively by HNF1A-MP_2 variants exhibits a severe disruption of the dimerization interface, resulting in the two HNF1A-MP_2 molecules no longer being properly connected (Fig. 12C). This structural separation likely impairs the ability of the homodimer to bind to DNA efficiently, further suggesting reduced transcriptional activity.

## Discussion

Understanding the molecular mechanisms underlying human disease is crucial for developing the most effective treatments and identifying early biomarkers of disease. Despite the significant prevalence of vascular complications in HNF1A-MODY individuals, comprehensive characterisation of the phenotypic alterations and the molecular mechanisms underlying these pathological changes remains elusive. A major constraint has been the lack of evidence of HNF1A expression in ECs. Here, we showed that HNF1A is expressed both in primary ECs and hiPSC-ECs. Although HNF1A levels are significantly lower in ECs than in the hepatocellular adenocarcinoma cell line HepG2, we showed that the reduction of HNF1A in ECs may mediate the phenotype/pathology observed in HNF1A-MODY patients. However, it should be noted that many of the mutations, especially the ones within the DNA-binding domain (DBD), have an effect on the protein functionality and not on the protein expression level.

Previously, we reported a differential HNF1A-dependent ECs permeability in normoglycemic conditions, emphasizing the role of HNF1A in the modulation of the ECs phenotype [16]. The general endothelial dysfunction leads to a dysregulated endothelial barrier function, which results in the development of atherosclerosis [43]. Given the increased intima-media thickness observed in HNF1A-MODY patients [15] under stabilized glucose levels, we performed all analyses in normoglycemic conditions. To pave the way for ECs-oriented research in HNF1A-MODY, we performed global transcriptome and proteome profiling. Both high-throughput analyses pointed out a robust HNF1A-dependent modulation of the ECs signalling, related to cytoskeleton, cell-cell interactions, and migration-related pathways (i.e. Rap1, Ras signalling). Our transcriptomic data are in line with previous transcriptome analysis of HNF1A-MODY patients’ alpha and beta cells, which showed alterations in cell-cell adhesion and ATP binding [9]. In the same patient islet cells, changes were related to the metabolism, cell cycle, and cell adhesion/motility [9]. Moreover, half of the up- and down-regulated genes were shown to overlap between the alpha and beta cells, highlighting a more general effect of the *HNF1A* mutations in different cell types.

The transcriptome profiling of hiPSC-ECs pointed out deregulation of AGE-RAGE signalling. The important role of advanced glycation end products (AGEs) and their receptor (RAGE) in the development of endothelial dysfunction and diabetes is well-established [44]. In ECs, the abnormal functioning of the AGE-RAGE axis is associated with the intensification of detrimental processes like oxidative stress and inflammation, along with abnormal motility [45]. In essence, endogenous AGEs produced in the course of hyperglycaemia induce endothelial damage and impaired healing [46], leading to canonical diabetes complications such as retinopathy [47]. Such changes are widely described in T2D [48]. Largely interconnected with RAGE signalling [49], we observed a significant profibrotic signature of *HNF1A*-mutated ECs. Interestingly, in a rat model of liver fibrosis, the reduction in *HNF1A* gene led to increased ECM deposition [50]. One of the crucial regulators of fibrosis, PAI-1, is increased during T2DM and linked with the development of macrovascular complications in T2DM and obesity [51]. High PAI-1 levels correlate with a reduction in insulin sensitivity [52], which was previously described in HNF1A-MODY patients [53]. Moreover, in accordance with our RNA-seq results, *HNF1A* knockdown was shown to enhance a proinflammatory phenotype through modulation of cytokines [54] or interferons [55]. Using our control-derived isogenic set of lines, we showed that mono- or biallelic mutations in *HNF1A* can lead to increased mRNA expression of IL-1B, IL-6, and IL-8. The proinflammatory phenotype was also confirmed with the RNA-seq analysis of the patient-derived isogenic set of lines, where an increase in the TLR signalling in the MP_1 hiPSC-ECs was observed. It is known that in addition to pathogens, TLRs detect host-derived danger-associated molecular patterns (DAMPs) released from injured or dying cells. Sustained or excessive TLR activation in vascular cells has been proposed to drive chronic low-grade inflammation, promoting endothelial dysfunction and ultimately contributing to cardiovascular disease [56]. The proinflammatory phenotype can have an additional consequence on the vascular glycocalyx structure, which was found to be decreased in the *HNF1A*-mutated cells from all three hiPSCs sets of lines.

Various studies showed that the reduction of the endothelial glycocalyx thickness occurs in disease conditions, including hypertension, atherosclerosis, and aging [57]. All these conditions have a common element of enhanced matrix stiffness. Whether glycocalyx protection or restoration can rescue EC integrity and prevent disease progression is still uncertain [58]. Observed diminished glycocalyx may lead to serious consequences for HNF1A-MODY individuals, like increased permeability both in basal and inflammatory or oxidative conditions [59]. Moreover, the plasma glycan profile alterations have been proposed as a diagnostic biomarker in HNF1A-MODY patients [60]. Shedding of endothelial glycocalyx impairs its structural integrity, leading to intensified permeability, which in turn leads to more glycocalyx shedding, resulting in a vicious circle [61]. Accordingly, in our previous work, we observed an increased ECs permeability in response to TNFα in *HNF1A*-mutated cells. The phenotype of ECs characterized by diminished glycocalyx and increased stiffness has already been observed in the early stages of T2D [62], which confirms the similar development of endothelial dysfunction in HNF1A-MODY and T2D. The impaired glycocalyx layer in hiPSC-ECs may explain the often diagnosed retinopathy in HNF1A-MODY patients [13].

In this study, we focused our attention on migration and mechanical features of ECs, because of our preliminary observation regarding vascular permeability. Moreover, the integrated global proteome and transcriptome analysis of the cells from the control-derived isogenic set of lines pointed at a possible dysregulation of the cell cytoskeleton. The same was also revealed when looking at DEGs that could be found in the BAC and in the HNF1A ChIP-seq putative target genes [32]. Moreover, the endothelial glycocalyx is directly linked to the cell cytoskeleton inside the cell. ECs migration is closely related to the regenerative capacity and ensures vessel integrity. Cellular adhesion, cytoskeleton remodelling, and signal transduction are all coordinated by mechanically integrated molecular processes [63]. Furthermore, elevated glucose levels and oxidative stress promote ECs migration in diabetic patients, particularly those with retinopathy [64]. Here we observed an increased migratory capacity of hiPSC-ECs with mono- and biallelic mutations in the *HNF1A* in comparison to the isogenic control. Other studies have observed that *HNF1A* silencing in hepatic cells also leads to increased migration [65], corroborating its essential role in cell migration. However, both patient-derived EC lines did not recapitulate the effect on cell migration observed in MAC and BAC. It is possible that the difference could not be seen in comparison to the control hiPSC-EC lines due to individual patient variability. Additionally, cellular stiffness could also affect cell migration. Of note, a significant difference in the patient set of hiPSCs between the two healthy controls regarding cellular stiffness was evident, showing that the effect of the genetic background could indeed have an impact on the analysed parameters. The discrepancies in the phenotype between control-derived isogenic cell lines and patient-derived ones were also observed in the case of cellular stiffness assessed by atomic force microscopy, which in this study correlates well with actin cytoskeleton polymerization. Thus, the differences in the migration between patient-specific and control-derived isogenic lines could also be attributed to the changes in cellular stiffness. According to most studies, the increased cellular stiffness leads to diminished migration [66] and can be an early marker of diabetes [62]. Importantly, when *HNF1A* levels in patient-derived lines were reduced by RNA interference, the cells had increased migratory capacity accompanied by reduced actin stress fibres. Moreover, comparing the HNF1A putative targets, identified by HNF1A ChIP-seq analysis in kidney organoids [32] and the DEGs in BAC, more than 300 common genes were found, part of which are implicated in actin organization and cell migration (Fig. 5C, Additional file 4: Table S3).

Using our isogenic patient-derived model, where *HNF1A* correction of the patient-specific mutation in MP_1, we successfully restored a decoration of the glycocalyx layer, proving that mutations in *HNF1A* affect the endothelial glycocalyx. However, genetic correction did not impact stress fibre organization and consequently did not influence cellular migration. Based on migration results from our control-derived isogenic cell lines, *HNF1A* correction should theoretically increase stress fibre formation and consequently reduce cellular migration, which contrasts with the results obtained using patient-derived isogenic cell lines. The explanation for these seemingly counterintuitive results lies in the origin of hiPSCs used in this study. The source of hiPSCs (healthy donors versus HNF1A-MODY patients) appears to influence endothelial stiffness itself, indicating that the effects of the diabetic environment may supersede the contribution of *HNF1A*. The phenotype observed in patient-derived and corrected hiPSC-ECs may be attributed to strong epigenetic effects that remain stable despite genetic correction and are likely linked to the diabetic state. The diabetic microenvironment, together with environmental factors, lifestyle, and dietary factors implicated in diabetes pathogenesis, can profoundly alter epigenetic states, potentially establishing cellular memory that persists in experimental models [67]. This epigenetic reprogramming is further influenced and reinforced by chronic hyperglycaemia, which in turn is a key inducer of increased aortic stiffness [62, 68]. Importantly, epigenetic memory can be retained not only in hiPSCs but may persist even after differentiation into specialized cell types, continuing the phenotypic influence of the cells from which the hiPSCs were originally derived [69, 70]. Cumulatively, our results suggest that both HNF1A proper function and the genetic background of the donors have an impact on the cytoskeletal organization (stiffness and actin fibres) and ECs migration in relation to HNF1A-MODY. The glycocalyx layer, which is connected to the cell cytoskeleton, is diminished in all three hiPSCs models, which might be an important endothelial dysfunction marker.

### Limitations of the study

Despite these important findings, this study has few limitations. First, although hiPSC-ECs provide valuable insights, the 2D culture system does not fully replicate the complexity of the 3D vascular environment. Future studies could consider employing 3D models or organ-on-a-chip platforms to more accurately mimic in vivo conditions. Additionally, while experiments were performed under normoglycemic conditions to isolate the direct effects of *HNF1A* mutations, investigating the endothelial response under hyperglycaemic conditions would offer a more comprehensive understanding of disease mechanisms in a diabetic context. Our study design may have excluded important endothelial responses to glucose fluctuations that are also relevant in disease progression. In summary, despite these limitations, our findings highlight actin and glycocalyx-based endothelial dysfunction in HNF1A-MODY and set the stage for future studies incorporating patient-specific isogenic lines, 3D culture systems, and variable glycaemic conditions to more comprehensively model disease mechanisms.

## Conclusions

In the current study, we have shown that HNF1A has a significant role in ECs homeostasis, affecting the cytoskeleton and several cell signalling cascades. The *HNF1A*-mutated ECs had an increased proinflammatory phenotype and a decreased glycocalyx layer. Additionally, the decreased *HNF1A* expression, through mutation or siRNA, leads to increased migration of hiPSC-ECs, which was mirrored by reduced actin stress fibres formation. Cumulatively, all these changes can lead to increased vascular permeability in normoglycemic conditions and subsequent endothelial dysfunction with vascular complications often diagnosed in HNF1A-MODY patients.

## Supplementary Information

Additional file 1: Figures S1-S13. Supplementary figures.

Additional file 2: Table S1, List of differentially expressed (DE) genes in MAC and BAC from control-derived isogenic set with log2fold change and padj values, in searchable Excel format. DE genes were used for subsequent GSEA analysis.

Additional file 3: Table S2, GSEA Reactome analysis of RNA-seq data from patient-derived isogenic set, and 100 most enriched pathways in searchable Excel format.

Additional file 4: Table S3, List of common genes between ChIP-seq of HNF1A from the study of Chan et al. [32] and differentially expressed genes in hiPSC-ECs with biallelic mutation in HNF1A (BAC line).

Additional file 5: Table S4, List of differentially expressed genes and differentially expressed proteins related to cell migration in both control-derived isogenic lines MAC and BAC.

Additional file 6: Supplementary methods, Detailed description of the methods used for the supplementary figures S1-S13.

## Data Availability

The global transcriptome data from the control-derived isogenic set have been deposited in the BioStudies database at EMBL-EBI under accession number E-MTAB-10404, and the patient-derived isogenic set under accession number E-MTAB-16548.Neli Kachamakova-Trojanowska (2026). Gene profiling of human iPS-ECs with monoallelic and biallelic mutation in HNF1A gene as compared to isogenic hiPS-ECs control cells. BioStudies, E-MTAB-10404. [https://www.ebi.ac.uk/biostudies/arrayexpress/studies/E-MTAB-10404], DOI: 10.6019/E-MTAB-10404; Array Express: E-MTAB-10404.Neli Kachamakova-Trojanowska (2026). RNA-seq of hiPSC-ECs from HNF1A-MODY patient and isogenic corrected lines. BioStudies, S-BSST2582. [https://www.ebi.ac.uk/biostudies/studies/S-BSST2582] , DOI: 10.6019/S-BSST2582; Array Express: E-MTAB-16548.The source code for RNA seq analysis is available in zenodo: Skoczek, D., Kloska, D., Targosz-Korecka, M., Szade, K., Biela, A., Hohendorff, J., Babincak, M., Kopacz, A., Malecki, M. T., Stepniewski, J., & Kachamakova-Trojanowska, N. (2026). Integrated transcriptome and proteome analyses unveil cytoskeletal alterations in an endothelial model of monogenic diabetes. W Genome Medicine. Zenodo. 10.5281/zenodo.18734217.The global proteome data were deposited in the ProteomeXchange Consortium with the dataset identifier PXD039471.

## Abbreviations

AFM: Atomic force microscopy
AGE: Advanced glycation end products
BAC: Biallelic clone
DAMPs: Danger–associated molecular patterns
DBD: DNA–binding domain
DEGs: Differentially expressed genes
DEPs: Differentially expressed proteins
ECs: Endothelial cells
ECM: Extracellular matrix
HD: Healthy donor
hiPSCs: Human induced pluripotent stem cells
hiPSC-ECs: ECs–endothelial cells derived from human induced pluripotent stem cells
HNF1A: Hepatocyte nuclear factor–1 homeobox A
MAC: Monoallelic clone
MODY: Maturity–onset diabetes of the young
MP: MODY patient
T1DM, T2DM: Type 1 diabetes mellitus, type 2 diabetes mellitus
TLR: Toll–like receptors
