# Supplementary material for: Integrated transcriptome and proteome analyses unveil cytoskeletal alterations in an endothelial model of monogenic diabetes
Source: Genome Med. 2026 Feb 27;18:38. doi: 10.1186/s13073-026-01615-z (PMC13049728; doi:10.1186/s13073-026-01615-z)
Supplement: Supplementary file 1 — Additional file 1: Figures S1-S13. Supplementary figures. [file 13073_2026_1615_MOESM1_ESM.pdf]

Integrated transcriptome and proteome analyses unveil cytoskeletal alterations in an endothelial model of monogenic diabetes

*Dawid Skoczek, Damian Kloska, Marta Targosz-Korecka, Krzysztof Szade, Artur Biela, Jerzy Hohendorff, Marian Babincak, Aleksandra Kopacz, Maciej T. Malecki, Jacek Stepniewski, Neli Kachamakova-Trojanowska**

Additional file 1: Fig. S1-S13

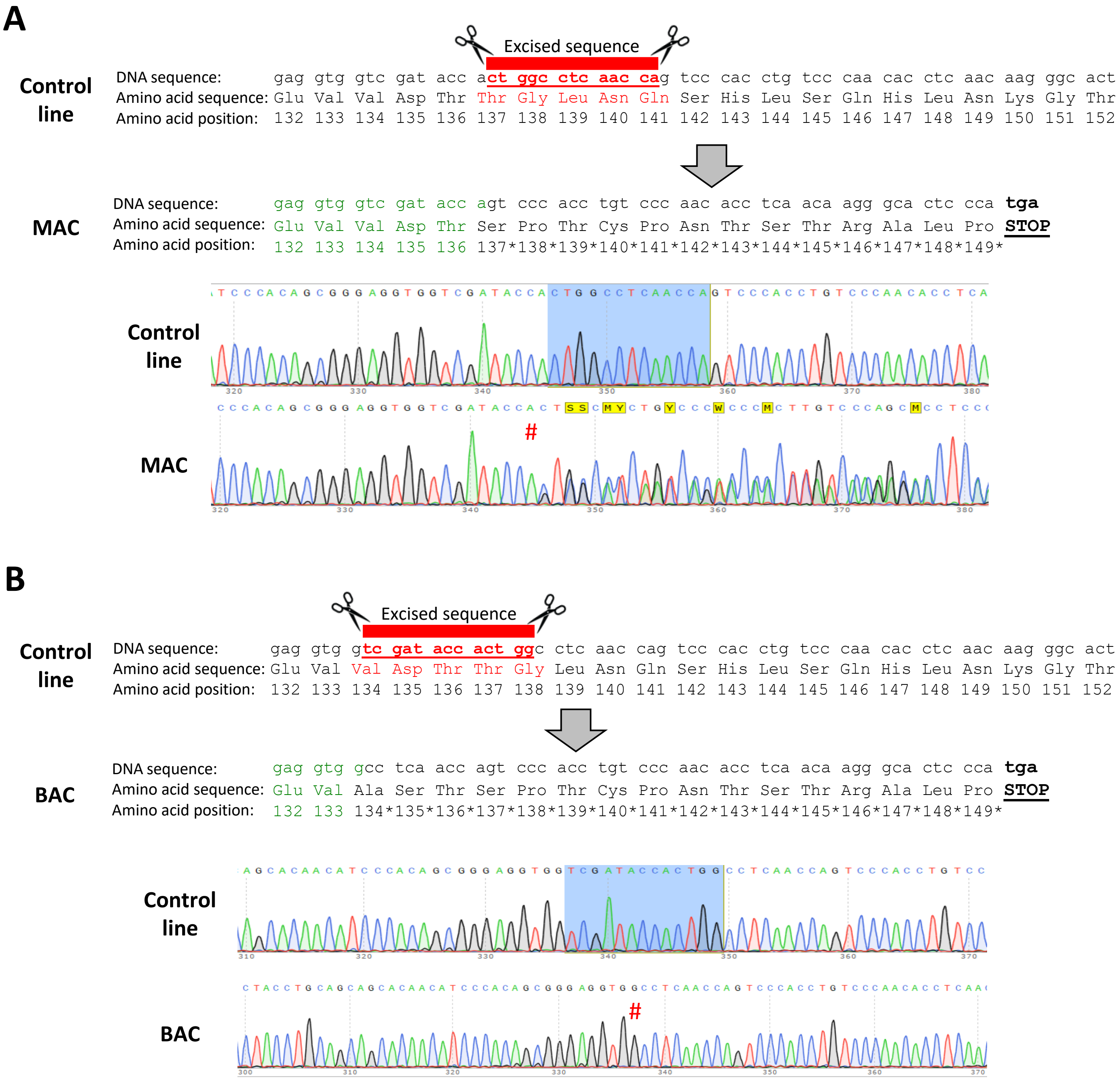

Fig. S1: Representation of the CRISPR/Cas9 introduced mutations in the isogenic HNF1A-MODY lines. MAC harbors monoallelic excision (A), whereas BAC biallelic (B). The mutated DNA sequences and the corresponding amino acids at the mutation sites are highlighted in red. The positions of the amino acids affected by these mutations are marked with asterisks (*). Sequences and amino acid positions that remained unaltered are highlighted in green. The Sanger sequencing results are shown for each mutated line, with the excised DNA fragments highlighted in blue. The last unchanged nucleotide before excision was marked with a hash (#).

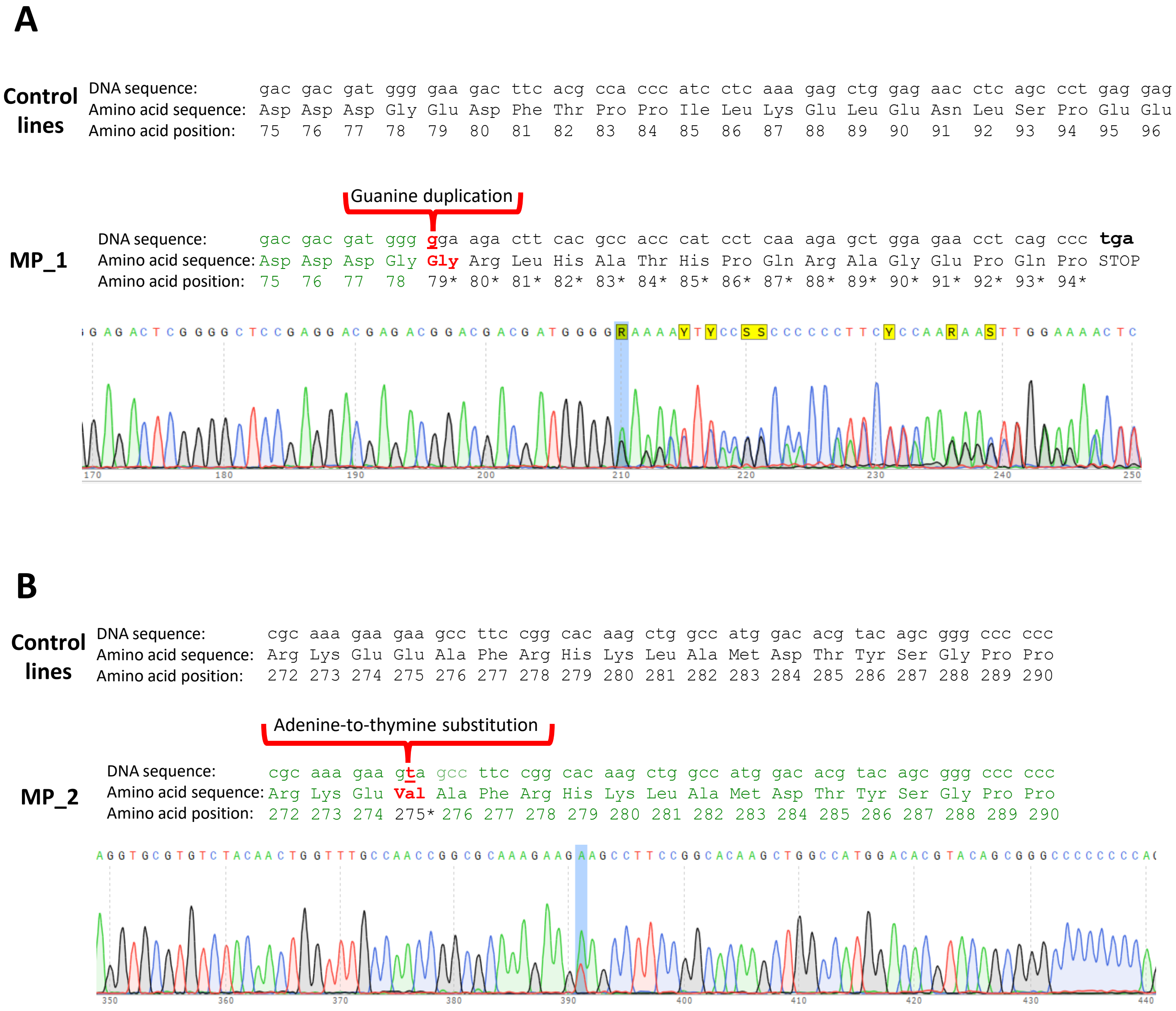

Fig. S2: Representation of the introduced mutations in the patient-specific HNF1A-MODY lines. MP_1 harbors a monoallelic c.235 guanine duplication (A), while MP_2 contains a monoallelic c.824 adenine-to-thymine substitution (B). The mutated DNA sequences and the corresponding amino acids at the mutation sites are highlighted in red. The positions of the amino acids affected by these mutations are marked with asterisks (*). Sequences and amino acid positions that remained unaltered are highlighted in green. For each mutated line, the Sanger sequencing results are shown, with the mutated nucleotides highlighted in blue.

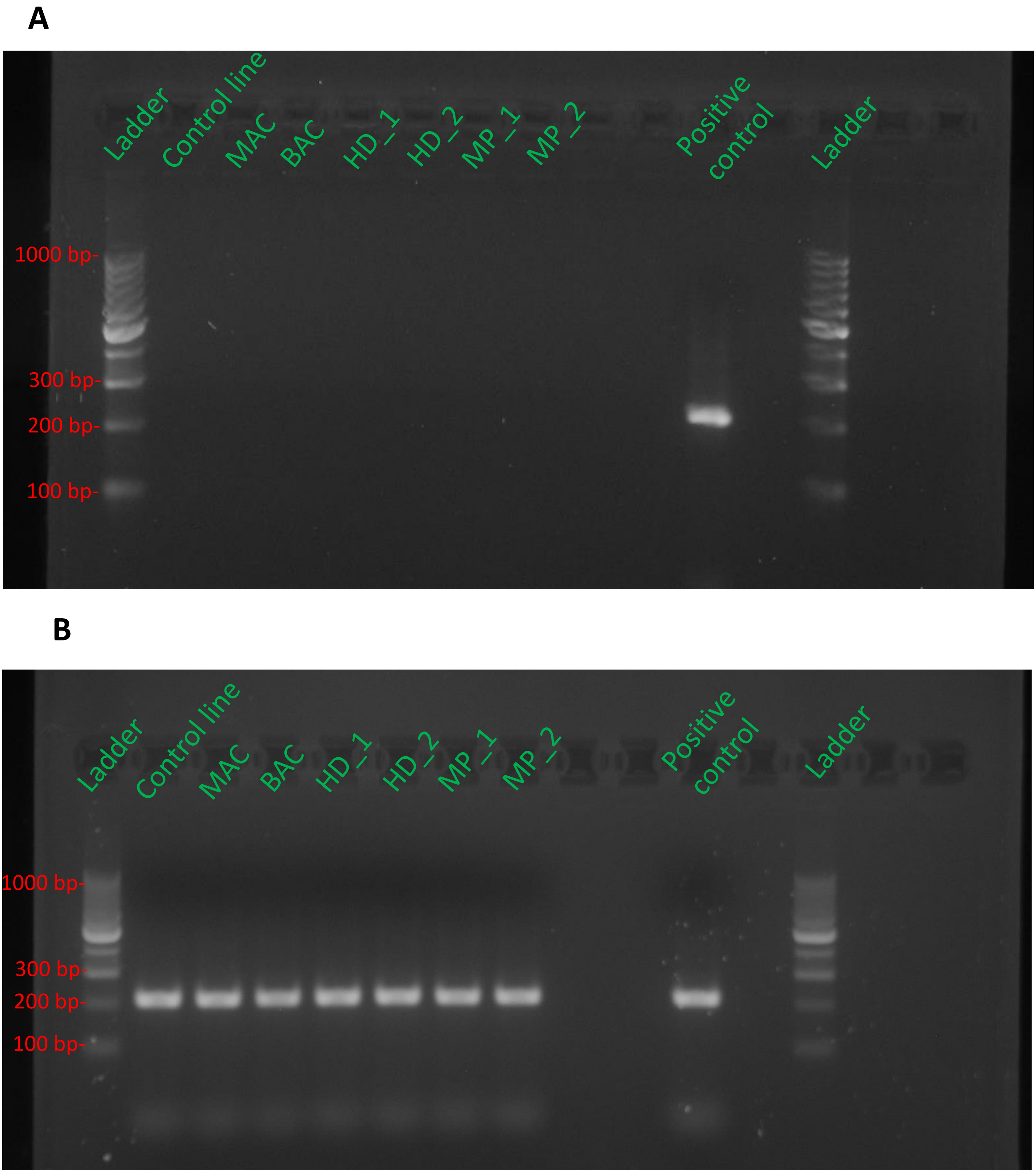

Fig. S3: (A) Analysis confirming the absence of reprogramming vector (Sendai virus) in the hiPSC lines. (B) The eEF2 gene was used as a housekeeping control. Positive control was obtained from PBMCs on day three of reprogramming.

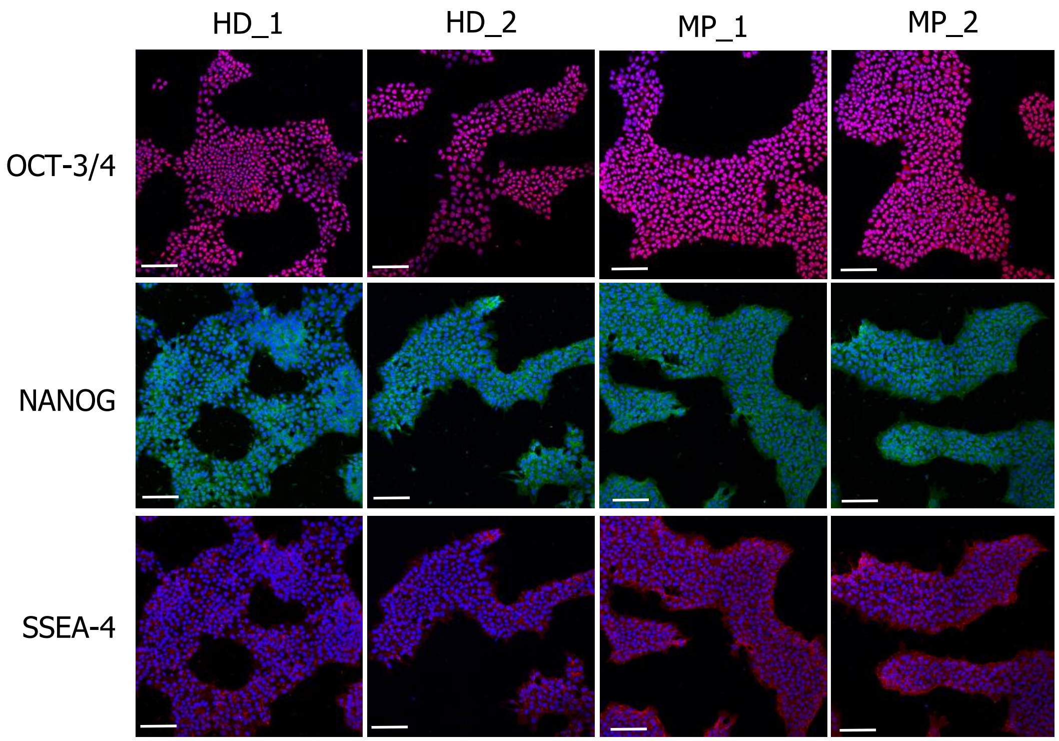

Fig. S4: Immunofluorescent staining of pluripotent markers of iPSCs derived from two healthy donors (HD_1 and HD_2) and two HNF1A-MODY patients (MP_1 and MP_2). OCT-3/4 is shown in red, NANOG in green, and SSEA-4 in red in the respective panels. Nuclei are stained with DAPI (blue). The images include a merged view showing co-localization with DAPI, indicating nuclear staining. Scalebar 100 µm.

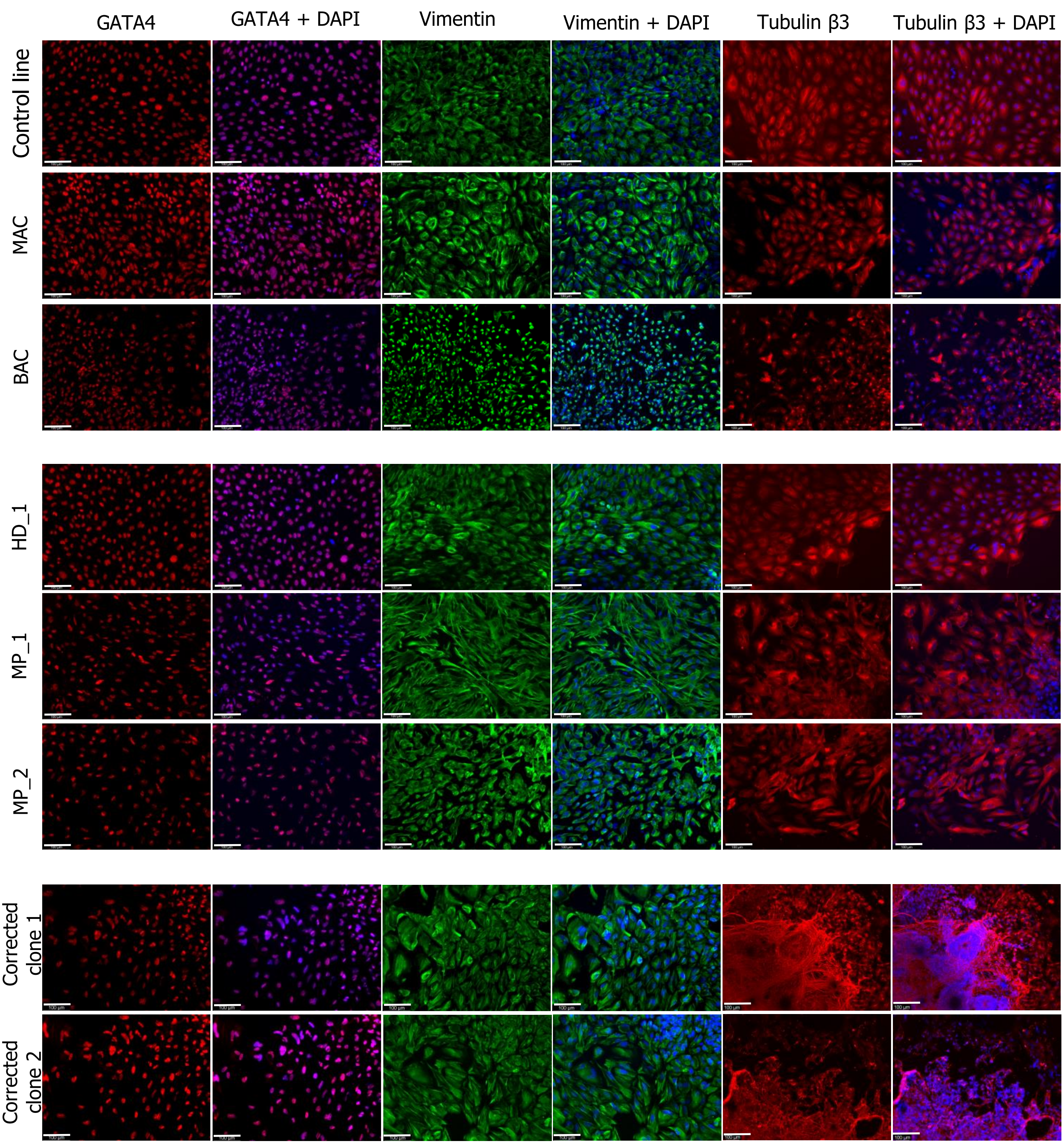

Fig. S5: Spontaneous *in vitro* differentiation of hiPSC lines via embryoid bodies and analyzed via immunocytochemistry for lineage-specific markers representative of the three embryonic germ layers. Endoderm is visualized using the marker GATA4 (red), mesoderm is visualized using the marker Vimentin (green), and ectoderm is visualized using the marker tubulin β-3 (also red). Nuclei are stained with DAPI. The images include fluorescence staining for each marker and a merged view showing co-localization with DAPI, indicating nuclear staining. Scalebar 100 µm.

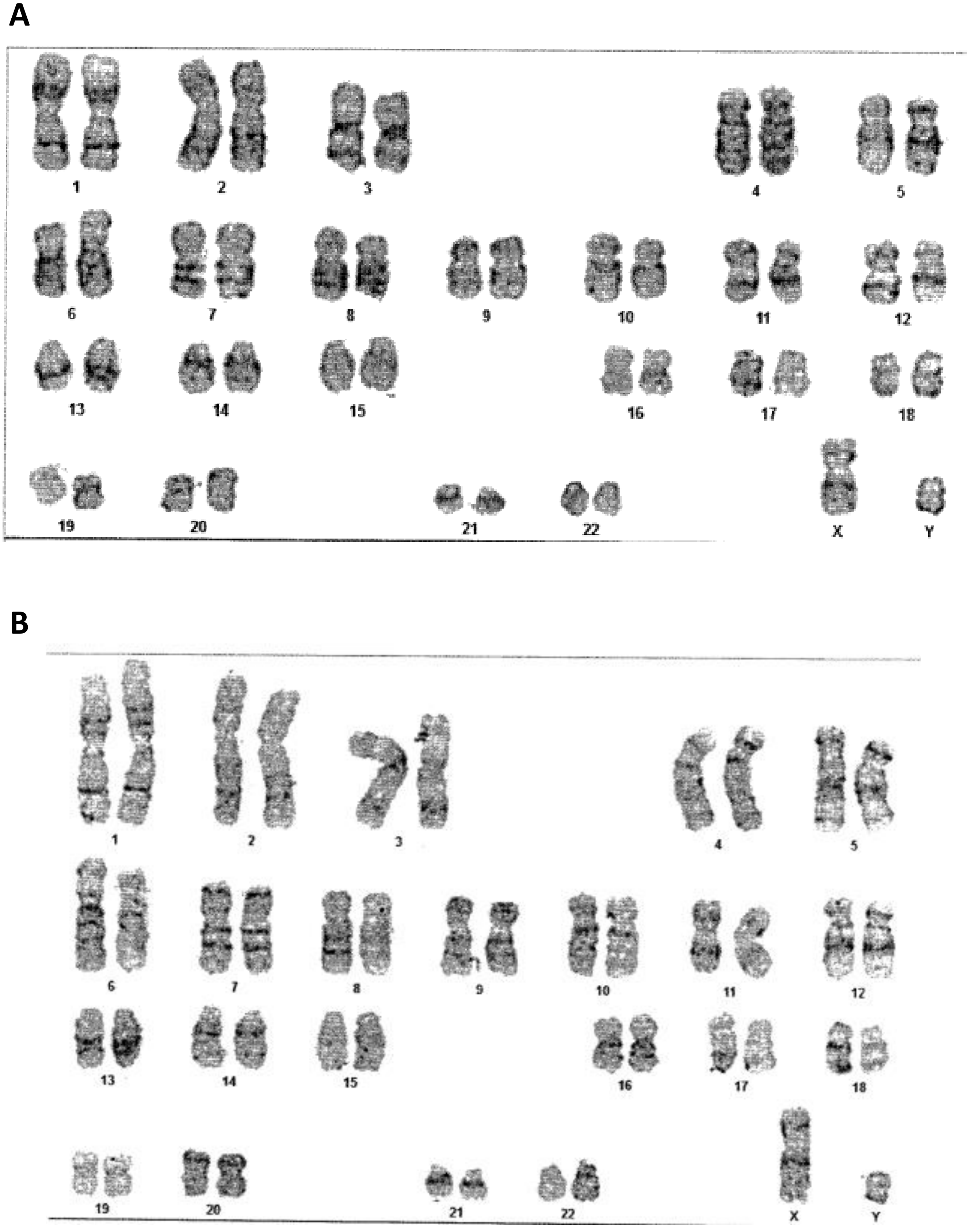

Fig. S6: Representative karyotype results of the control-derived isogenic hiPSCs lines MAC (A) and BAC (B). Both karyotypes demonstrate a correct chromosome content and structure.

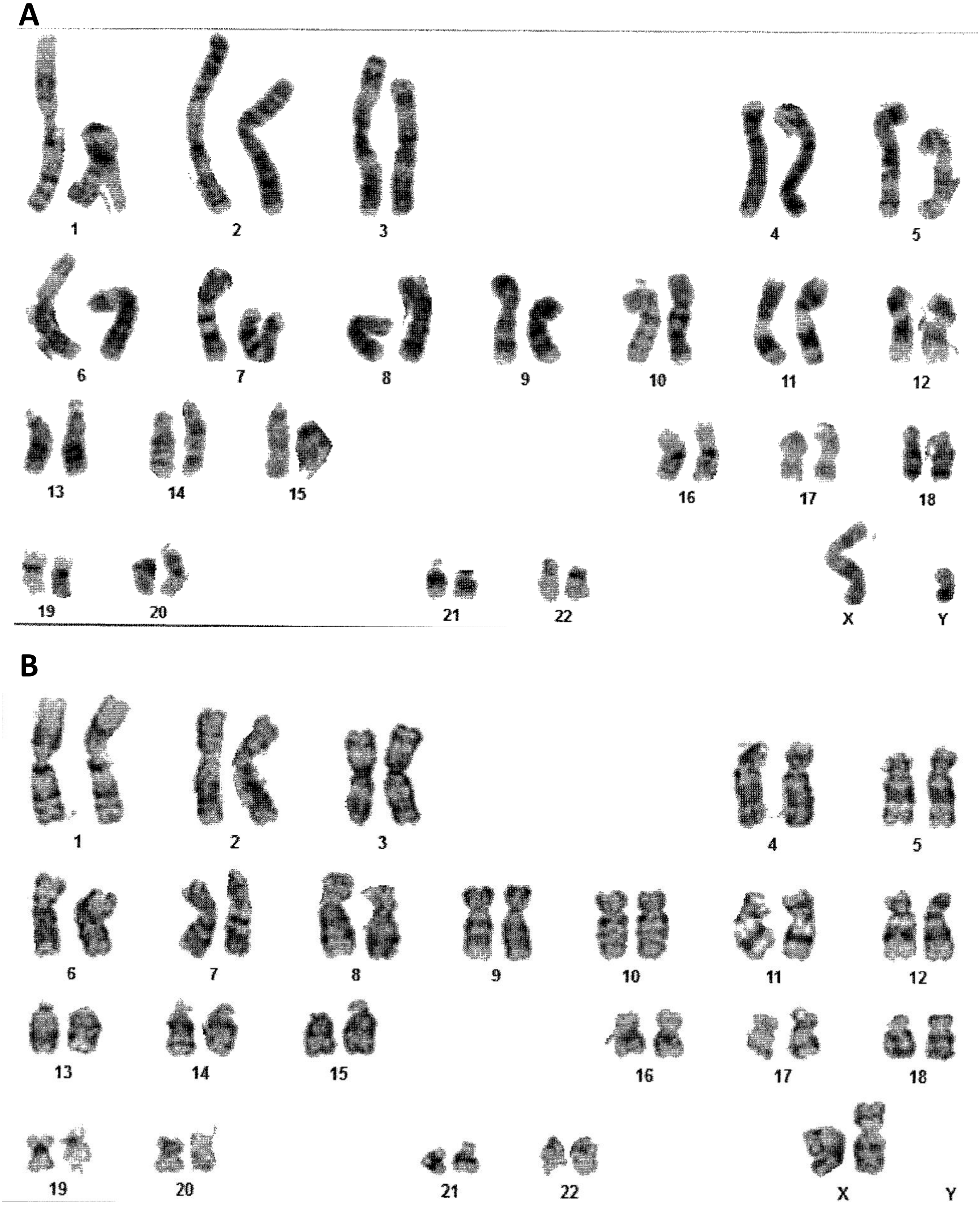

Fig. S7: Representative karyotype results of patient-specific hiPSCs lines MP_1 (A) and MP_2 (B). Both karyotypes demonstrate a correct chromosome content and structure.

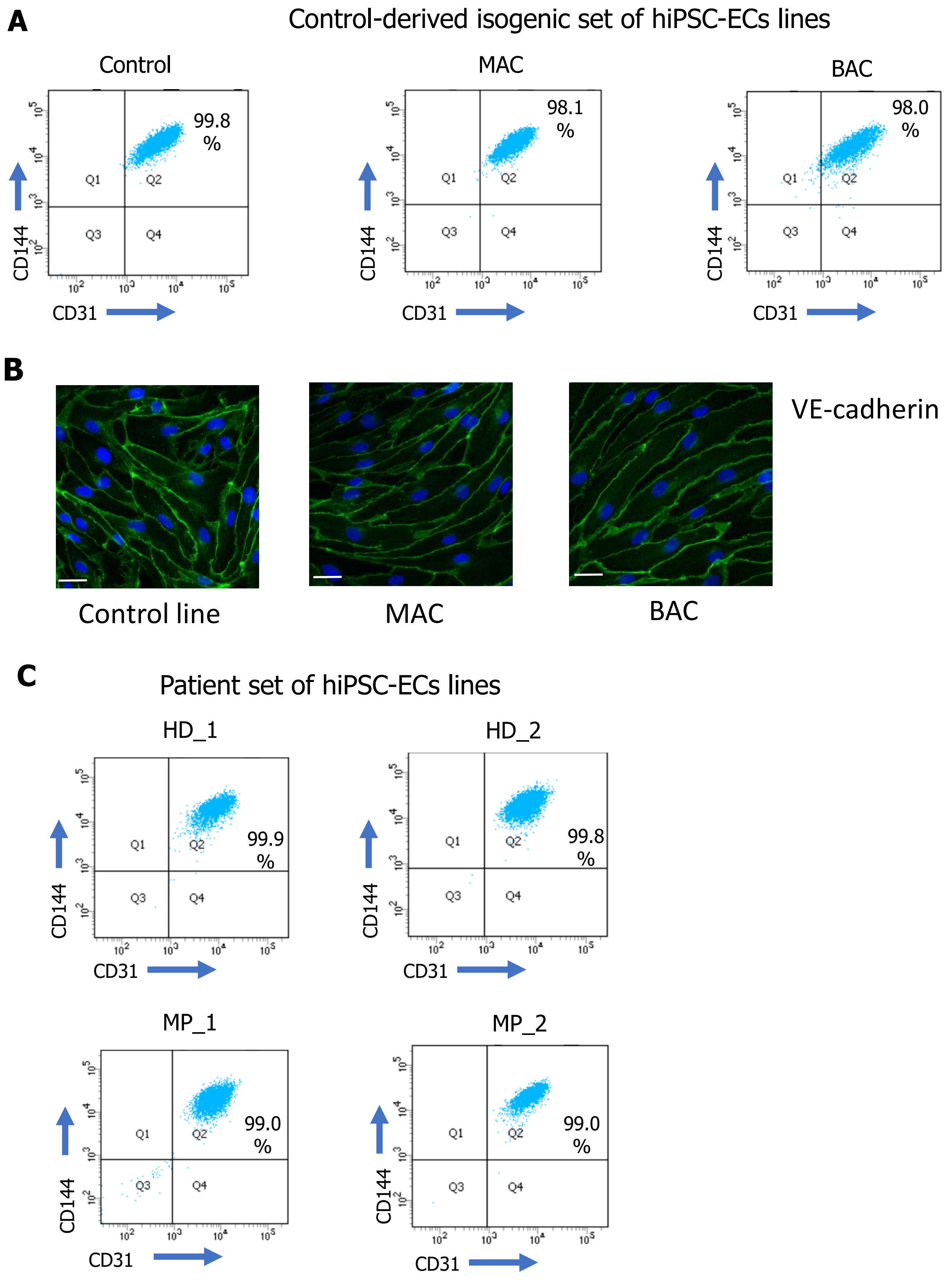

Fig. S8: (A) Representative plots with expression of VE-cadherin (CD144) and PECAM-1 (CD31) of hiPSC-ECs from the control-derived isogenic set assessed by flow cytometry. (B) Immunofluorescent staining of VE-cadherin (green) of hiPSC-ECs from the isogenic set. (C) Representative plots with the expression of VE-cadherin (CD144) and PECAM-1 (CD31) of hiPSC-ECs from the patient set assessed by flow cytometry. Scalebar 20 µm.

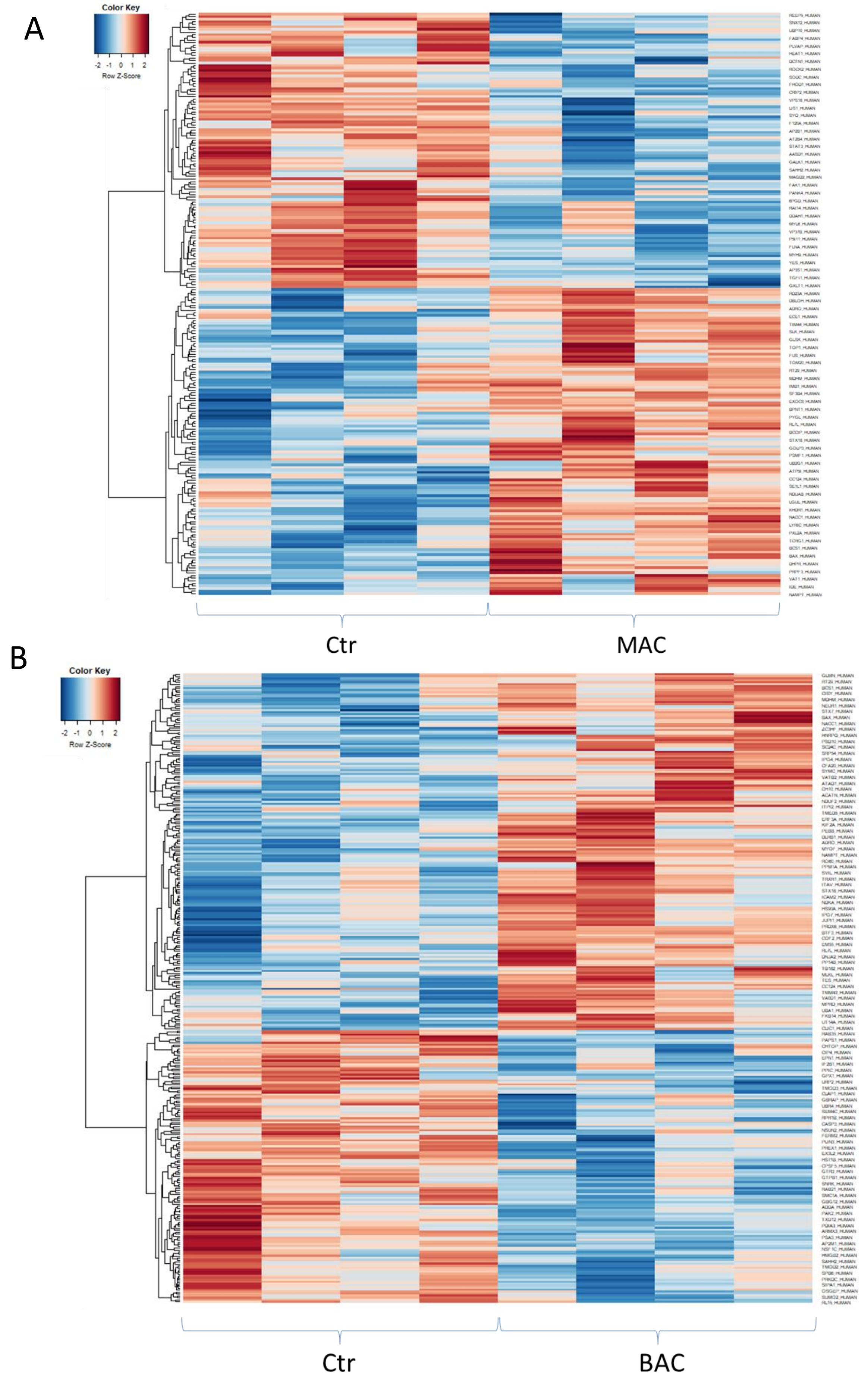

Fig. S9: Heatmap and cluster analysis of DEPs from hiPSC-ECs isogenic set. MAC vs unmutated (A) and BAC vs unmutated hiPSC-ECs (B). Each line in the heatmap represents the mean fold-change of up- (red) or down-regulated (blue) proteins. The results were obtained from 4 independent differentiations.

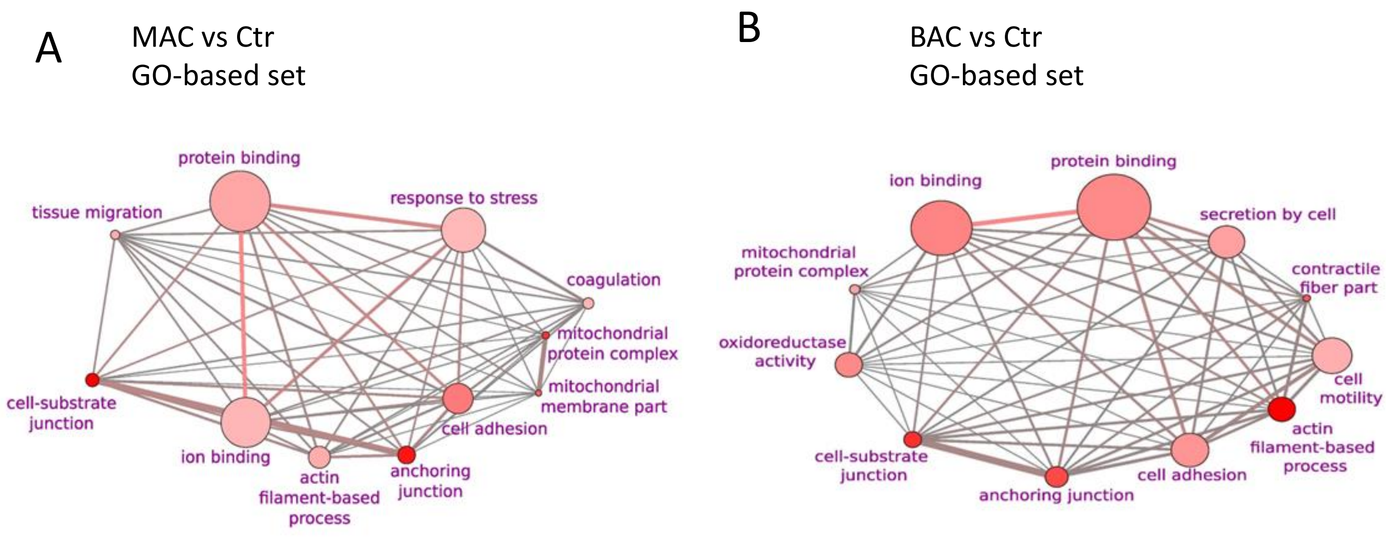

Fig. S10: Proteomic analysis of HNF1A-MODY hiPSC-ECs. Enrichment analysis of DEPs in GO–based sets of MAC (**A**) and BAC (**B**) created with ConsensuspathDB. Each node represents a separate concept for which size and p-value are encoded as node size and node colour, respectively. The edge colour encodes the number of shared members.

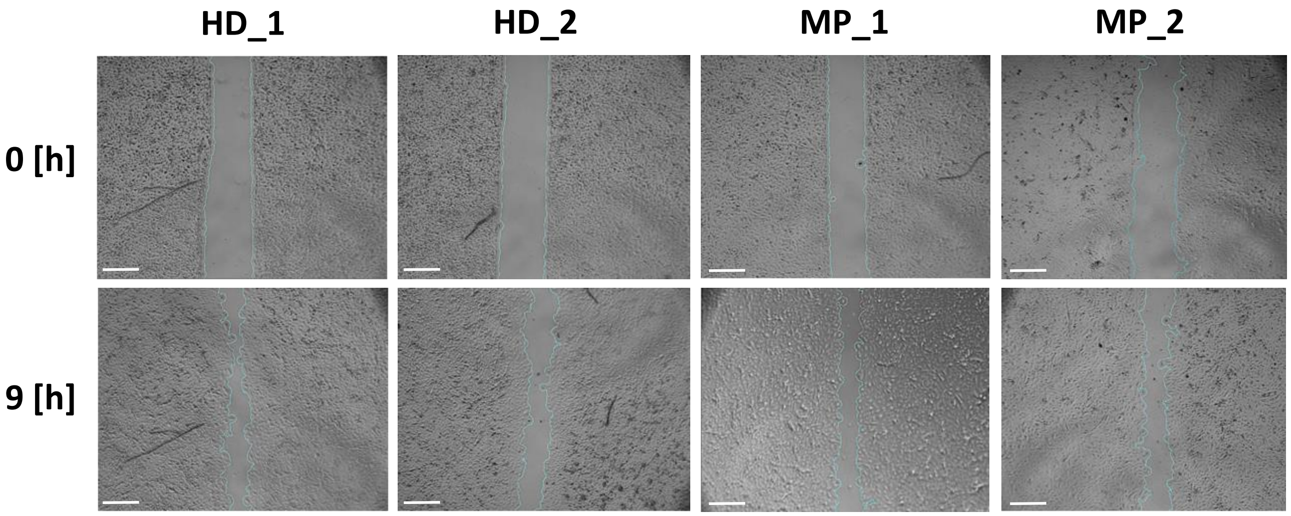

Fig. S11: Time-lapse images (time points: 0h and 9h) of scratch closure of healthy (HD_1 and HD_2) and HNF1A-MODY patient (MP_1 and MP_2) derived hiPSC-ECs. Scalebar 500 µm.

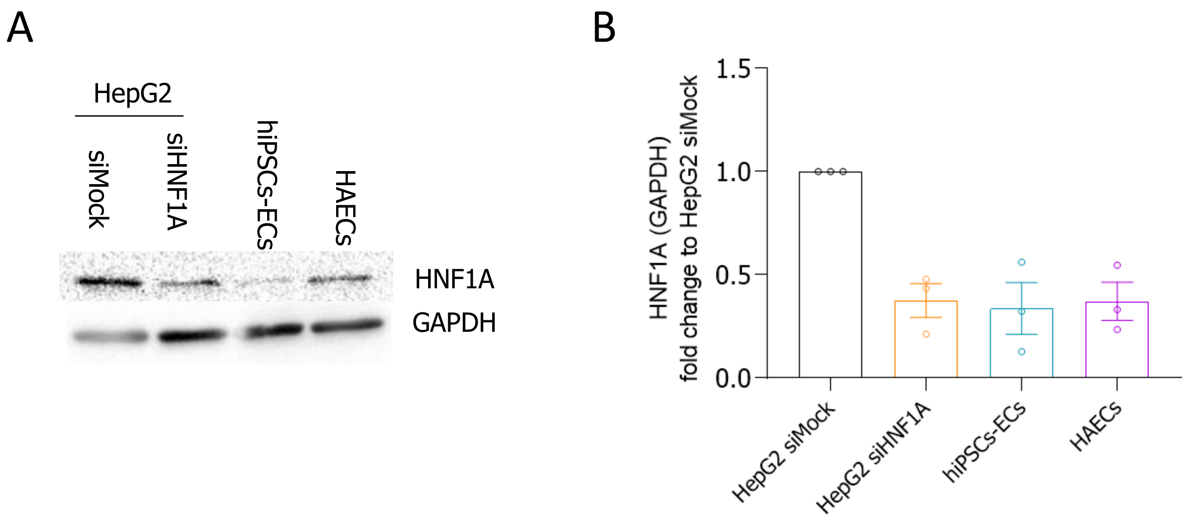

Fig. S12: (A) Representative western-blot analysis of HNF1A protein level in HepG2 cells transfected with siMock and siHNF1A, hiPSC-ECs and HAECs. (B) Densitometry analysis of the Western blot. GAPDH was used as a loading control. The analysis was performed from at least three independent differentiation experiments.

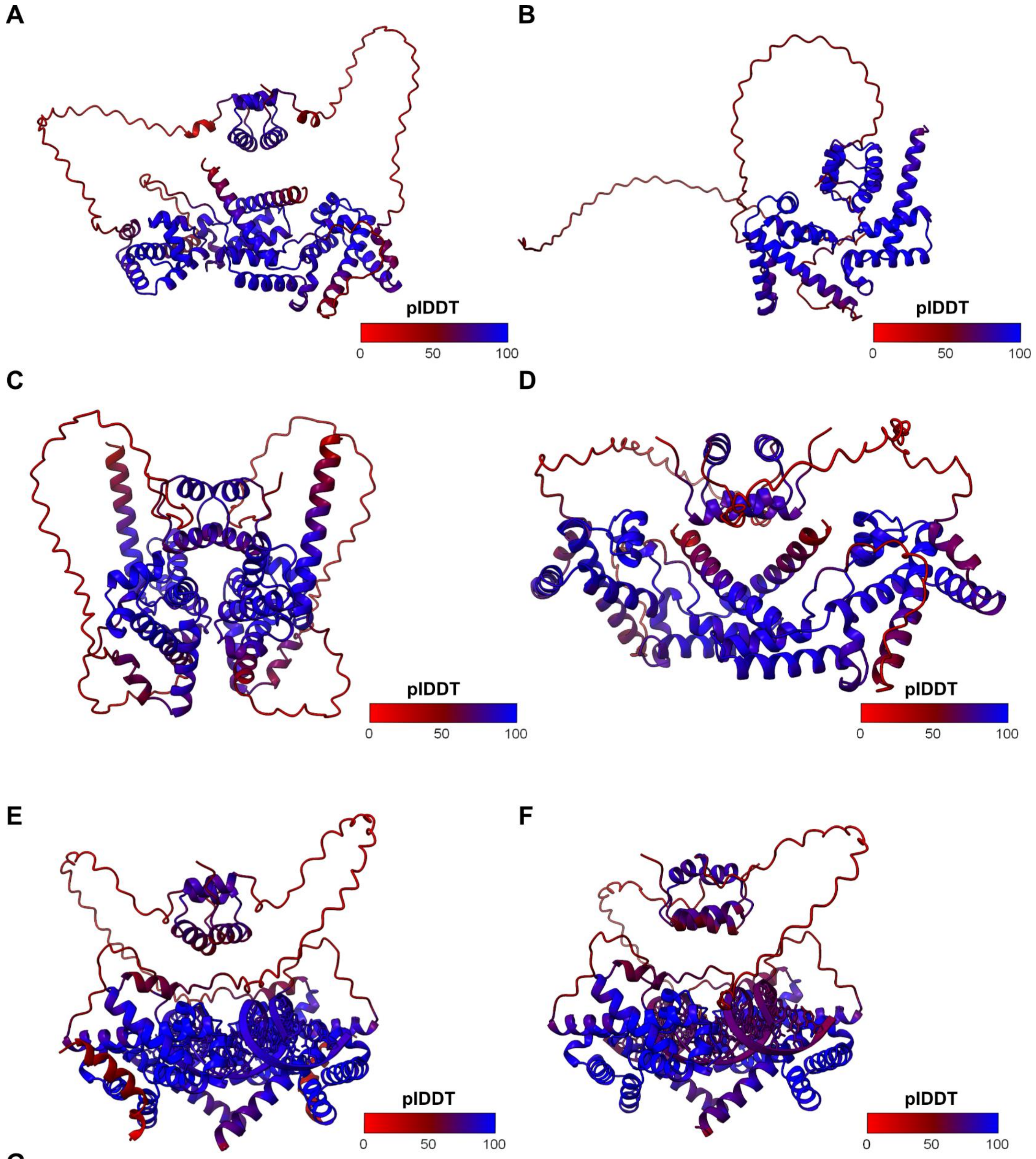

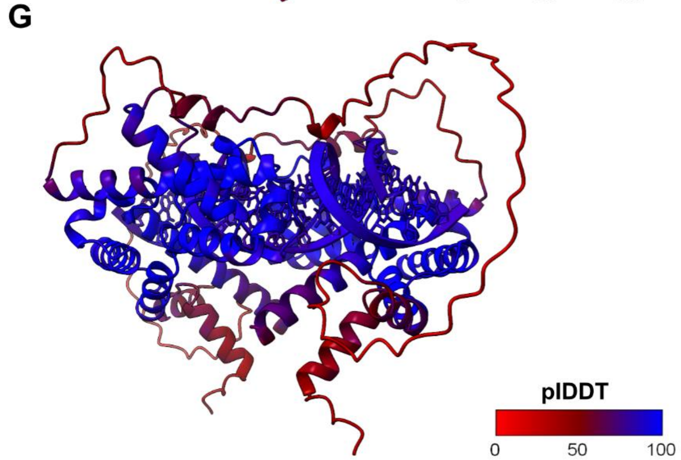

Fig. S13: Overall, AlphaFold 3.0 predicted structures of: (A) HNF1A WT dimer, (B) HNF1A WT-MP1 dimer, (C) HNF1A WT-MP2 dimer, (D) HNF1A MP2-MP2 dimer, (E) HNF1A WT dimer with bound dsDNA, (F) HNF1A WT-MP2 dimer with dsDNA, (G) HNF1A MP2-MP2 dimer with dsDNA. Dimers coloured according to the prediction score (pIDDT). In all cases, protein core seems to be predicted very confidently. The dsDNA prediction is worst in case HNF1A MP2 homo-dimer (F). Unstructured regions of the protein were hidden to enhance figure clarity.
