# Supplementary material for: Integrated transcriptome and proteome analyses unveil cytoskeletal alterations in an endothelial model of monogenic diabetes
Source: Genome Med. 2026 Feb 27;18:38. doi: 10.1186/s13073-026-01615-z (PMC13049728; doi:10.1186/s13073-026-01615-z)
Supplement: Supplementary file 4 — Additional file 4: Table S3, List of common genes between ChIP-seq of HNF1A from the study of Chan et al. [32] and differentially expressed genes in hiPSC-ECs with biallelic mutation in HNF1A (BAC line). [file 13073_2026_1615_MOESM4_ESM.docx]

AAK1

ABTB2

ACAD9

ACBD5

ACER2

ACTN1

ACTR1B

ACVR1

ADAMTSL1

AFAP1L2

AFG3L2

AGFG2

ALG11

ANAPC13

ANKRD13A

ANKRD17

ANP32A

AQP1

ARGLU1

ARHGEF2

ARRB2

ASB9

ATP1B1

ATP5G3

AXL

BDH1

BIRC2

BMF

BNIP3L

BRAT1

BRWD1

BTBD3

BTF3

BTG1

BUD31

C10orf11

C11orf31

C4orf47

C5orf30

C6orf48

C9orf72

CALM2

CALU

CARD11

CCDC47

CCDC88C

CCM2

CD164

CDC6

CDC7

CDK17

CDKN1A

CDKN1B

CDKN1C

CEP170

CEP57

CHMP1B

CLK1

CNBP

CNN3

COG4

COL13A1

COL1A2

COX20

CREBBP

CREM

CS

CTDSP2

CXXC5

DAPK2

DBP

DCC

DDX5

DDX6

DGCR14

DKK3

DLAT

DNAJB4

DNMT1

DOCK6

DYNLL1

DYNLL2

EBF3

EDNRB

EEF1A1

EFNB2

EGR1

EIF1

EIF4A2

ENC1

EPB41L5

EPHA5

ETV5

EXD3

EXOSC1

EXOSC8

EZH2

FADS1

FAM117A

FAM171A2

FAM213B

FAM216A

FAT1

FBLN1

FDFT1

FHL2

FKBP14

FKBP7

FLRT3

FOXN3

FRMD4A

FZD1

GAB1

GABARAP

GATAD2B

GFOD1

GMFB

GMNN

GPKOW

GREB1L

GSE1

GSK3B

HERPUD1

HIF1AN

HIST1H2BC

HIST1H2BD

HK2

HMGCS1

HN1

HNRNPL

HOXA10

HOXA5

HOXA9

HOXB4

HOXC6

HOXC9

HS3ST3A1

HS3ST3B1

HS6ST1

HSP90AA1

HSP90B1

ID2

IDI1

IFT57

INA

ING4

IRF2BP2

IVNS1ABP

JAK1

KDM3A

KIF1B

KIF22

KLHDC2

KLHL24

LAMTOR3

LBR

LDLRAP1

LIMA1

LMNB1

LOXL1

LRIG3

LRP3

LSAMP

MAFK

MAP3K11

MAPRE1

MBTD1

MCL1

MCM4

MECR

MEIS2

METTL9

MIR100HG

MIS18A

MLXIP

MN1

MSH6

MSX2

MYO18A

MYO1B

NAA20

NAA25

NCALD

NCAM2

NDUFA11

NDUFB2

NDUFS7

NMD3

NMT2

NOTCH4

NPY1R

NR2F1

NR2F2

NRBP1

NRG1

NUMA1

NXN

P4HA1

PALM2

PAPSS1

PBX1

PCBP4

PDCD11

PFKFB3

PGAM1

PGAP1

PGRMC2

PHLPP1

PHRF1

PIK3C2B

PJA2

PKIA

PKP4

PLCB1

PLXNA2

PLXND1

POLR1A

POLR2F

PPM1L

PPP1R14B

PPP1R15A

PRICKLE2

PSIP1

PSMA3

PSMA6

PYGL

PYGO1

RBL2

RDX

RFTN2

RFX8

RGMB

RGS3

RHOBTB3

RND3

RPL10A

RPL18A

RPL21

RPL22

RPL27A

RPL30

RPL39

RPS12

RPS18

RPS2

RPS23

RPS25

RPS3

RPS5

RPS6KA2

RRAGA

RTN4

RUFY3

RUSC2

SACS

SASH1

SAT1

SCD

SEC22B

SEC61G

SEMA3C

SERP1

SETBP1

SETX

SHC2

SIPA1L1

SKP1

SLC25A30

SLC25A37

SLC29A1

SLC38A1

SLC38A2

SLC45A4

SLC7A11

SLIRP

SNHG8

SNRNP200

SOX11

SOX5

SPRED1

SPRY2

SPTBN1

STC2

STRN4

STT3B

STXBP1

SVIL

TACR1

TAF13

TAF1D

TBX3

TM9SF3

TMED2

TMEM126A

TMPO

TMSB4X

TMX1

TNC

TNPO1

TNRC18

TOPBP1

TPP2

TRAF7

TRIM36

TRIO

TSC22D1

TUBA1C

TUBB2B

TUBB3

TXNIP

UBE2N

UBN2

UFM1

UNC5B

USO1

USP1

USP28

VEGFA

WASF1

WBP2

WWC1

YAP1

ZIC2

ZIC3

ZNF175

ZNF251

ZNF503

ZSWIM6
