# Supplementary material for: Integrated transcriptome and proteome analyses unveil cytoskeletal alterations in an endothelial model of monogenic diabetes
Source: Genome Med. 2026 Feb 27;18:38. doi: 10.1186/s13073-026-01615-z (PMC13049728; doi:10.1186/s13073-026-01615-z)
Supplement: Supplementary file 5 — Additional file 5: Table S4, List of differentially expressed genes and differentially expressed proteins related to cell migration in both control-derived isogenic lines MAC and BAC. [file 13073_2026_1615_MOESM5_ESM.docx]

**DEG in MAC vs control related to cell migration**

| Gene Name | log2FC (Ctr vs MAC) | adj. p value (Ctr vs MAC) |
| --- | --- | --- |
| TAC1 | -5,372712144 | 1,39797E-06 |
| SELE | -3,901668553 | 3,68994E-10 |
| GPC4 | -2,541380205 | 0,00021556 |
| SIX1 | -1,886430142 | 0,008886445 |
| SEMA5A | -1,809802681 | 1,08244E-14 |
| NKX2-3 | 1,754052523 | 0,000811028 |
| FGF2 | 1,784751503 | 1,40836E-10 |
| SH3RF2 | 1,786036431 | 0,005168308 |
| APCDD1 | 1,786611735 | 0,028412796 |
| FOXF1 | 1,792078938 | 0,000220306 |
| SULF1 | 1,834618322 | 0,000869923 |
| PPARG | 1,83914732 | 1,60984E-05 |
| HOXA5 | 1,867646858 | 6,37471E-11 |
| ANGPT1 | 1,965938839 | 0,008098854 |
| HOXA7 | 2,075764978 | 0,00047124 |
| HOXB9 | 2,282061512 | 1,49045E-11 |
| IGF2 | 2,614217886 | 0,000917708 |
| COL3A1 | 2,721778373 | 6,38264E-12 |
| POSTN | 3,26816836 | 1,20351E-18 |

**DEG in BAC vs control related to cell migration**

| Gene Name | log2FC (Ctr vs BAC) | adj. p value (Ctr vs BAC) |
| --- | --- | --- |
| FAM107A | -4,492049988 | 1,04755E-08 |
| SOX1 | -3,734997209 | 0,023794883 |
| IL33 | -3,093217684 | 0,006704808 |
| ARC | -2,871682964 | 0,043526348 |
| NOS3 | -2,408560186 | 4,43997E-27 |
| BST2 | -2,382539905 | 0,013861113 |
| GPC5 | -2,033950218 | 0,000215196 |
| TBXA2R | -1,99750388 | 1,16449E-21 |
| PLCG2 | -1,980827841 | 0,001953242 |
| SIX1 | -1,980558772 | 0,00059669 |
| MCTP1 | -1,856074586 | 1,48396E-14 |
| AQP1 | -1,780164752 | 0,001977725 |
| ENPP2 | -1,761527584 | 4,40313E-16 |
| SEMA5A | -1,702915919 | 7,92139E-12 |
| NTN4 | -1,63808788 | 3,16676E-14 |
| CLDN5 | -1,611516813 | 0,001034166 |
| GPC4 | -1,587094676 | 2,9518E-11 |
| IGFBP3 | -1,505265662 | 0,005442925 |
| WWC1 | 1,503423686 | 0,004148929 |
| MMP10 | 1,509603862 | 7,2203E-09 |
| EDNRB | 1,522222049 | 0,002088927 |
| PLAT | 1,554589038 | 2,44724E-10 |
| PDGFC | 1,568439588 | 2,83031E-15 |
| SORL1 | 1,604817671 | 0,002120467 |
| PAK1 | 1,612680633 | 2,27027E-07 |
| SRPX2 | 1,648194535 | 9,09211E-25 |
| SERPINE1 | 1,651593753 | 0,000180195 |
| ITGA11 | 1,683663698 | 4,46592E-06 |
| HOXB9 | 1,698393589 | 2,04756E-06 |
| EPHB2 | 1,739484895 | 0,023116645 |
| TNFSF4 | 1,746233323 | 0,01486628 |
| APCDD1 | 1,778216202 | 0,024634683 |
| JAM2 | 1,816335492 | 0,020691687 |
| FAT3 | 1,832197154 | 0,000789188 |
| VEGFA | 1,884724882 | 6,44644E-06 |
| DCLK1 | 1,932818573 | 2,67208E-09 |
| NAV3 | 2,018561246 | 5,41988E-09 |
| EGFR | 2,090383094 | 2,60431E-11 |
| CD44 | 2,159653076 | 0,004580723 |
| DOCK10 | 2,190793205 | 5,92109E-08 |
| FGF5 | 2,219485001 | 0,018139555 |
| PPARG | 2,2451919 | 5,41988E-09 |
| LPAR1 | 2,280232914 | 0,001954226 |
| SPOCK1 | 2,372753855 | 2,31096E-10 |
| LEF1 | 2,378233432 | 0,001985637 |
| SEMA3C | 2,459405801 | 0,002668801 |
| FLRT3 | 2,560432113 | 3,13244E-13 |
| VCAN | 2,626172423 | 1,29279E-07 |
| TNC | 2,634669347 | 5,63707E-18 |
| GFRA1 | 2,820568544 | 0,000228522 |
| IGF1 | 2,926209685 | 0,000270117 |
| PTPN22 | 2,94375968 | 3,63496E-06 |
| COL3A1 | 2,963421902 | 2,55152E-14 |
| COL1A1 | 3,211384854 | 2,59575E-35 |
| FOXF1 | 3,223833377 | 6,24664E-15 |
| IGFBP5 | 3,24660436 | 2,64857E-63 |
| HAND2 | 3,317130394 | 1,15785E-06 |
| GREM1 | 3,750249388 | 5,47521E-06 |
| RELN | 4,224698557 | 2,14803E-10 |
| EDNRA | 4,920654703 | 3,11955E-05 |

**DEP in MAC vs control related to cell migration**

| Protein Name | p value (Ctr vs MAC) | log2FC (Ctr vs MAC) | FC (Ctr vs MAC) |
| --- | --- | --- | --- |
| GSK3B | 0,002458357 | -1,278582096 | -2,426004281 |
| NOS3 | 0,002525849 | -0,616275311 | -1,532912459 |
| DYSF | 0,000373708 | -0,412740707 | -1,331212336 |
| PLVAP | 0,067818082 | -0,385807514 | -1,306590916 |
| PTN23 | 0,04274564 | -0,361998081 | -1,285204628 |
| NRP1 | 0,025877633 | 0,364035606 | 1,287021011 |

**DEP in BAC vs control related to cell migration**

| Protein Name | p value (Ctr vs BAC) | log2FC (Ctr vs BAC) | FC (Ctr vs BAC) |
| --- | --- | --- | --- |
| VAV3 | 0,00395718 | -1,588418166 | -3,007194467 |
| PLVAP | 0,01809698 | -1,0353899 | -2,049667511 |
| PREX1 | 0,040198608 | -0,964408398 | -1,951263208 |
| SEM4C | 0,045527339 | -0,82247448 | -1,768436577 |
| HPSE | 0,09521727 | -0,751790365 | -1,683881208 |
| NOS3 | 0,000402762 | -0,736914953 | -1,666608164 |
| LIMC1 | 0,066620075 | -0,715461731 | -1,642008654 |
| TYB4 | 0,010369581 | -0,702580452 | -1,627413036 |
| URP2 | 0,099802058 | -0,622851372 | -1,539915696 |
| ITA9 | 0,026167555 | -0,57461071 | -1,48927555 |
| MYH10 | 0,007630819 | -0,570421219 | -1,484957065 |
| HMGB2 | 0,0949087 | -0,543220997 | -1,457222319 |
| GRN | 0,020081272 | -0,483953476 | -1,398570981 |
| CXCR4 | 0,093266358 | -0,441361904 | -1,357885567 |
| ZO1 | 0,055630718 | -0,436261177 | -1,353093165 |
| MMRN2 | 0,019802974 | -0,411893845 | -1,330431143 |
| VGFR1 | 0,055640717 | -0,389348507 | -1,309801789 |
| RCC2 | 0,093351003 | -0,360492229 | -1,283863861 |
| A4 | 0,040534486 | 0,382712841 | 1,303791198 |
| DPYL3 | 0,046683327 | 0,386281013 | 1,307019815 |
| VAV2 | 0,076872199 | 0,454726219 | 1,370522696 |
| ITAV | 0,092239229 | 0,461256981 | 1,376740813 |
| TENS3 | 0,049470585 | 0,468394121 | 1,383568546 |
| CD151 | 0,014506501 | 0,582628886 | 1,497575653 |
| PODXL | 0,055275919 | 0,608664513 | 1,524847021 |
| EM55 | 0,062019567 | 0,625299454 | 1,542530968 |
| PALLD | 0,031031685 | 0,676732063 | 1,598514758 |
| CD9 | 0,034381304 | 0,750279903 | 1,682119154 |
| EGLN | 0,026736331 | 0,783351898 | 1,721125022 |
| CD44 | 0,019981807 | 1,126777172 | 2,183703787 |
