# Supplementary material for: Integrated transcriptome and proteome analyses unveil cytoskeletal alterations in an endothelial model of monogenic diabetes
Source: Genome Med. 2026 Feb 27;18:38. doi: 10.1186/s13073-026-01615-z (PMC13049728; doi:10.1186/s13073-026-01615-z)
Supplement: Supplementary file 6 — Additional file 6: Supplementary methods, Detailed description of the methods used for the supplementary figures S1-S13. [file 13073_2026_1615_MOESM6_ESM.docx]

**Integrated transcriptome and proteome analyses unveil cytoskeletal alterations in an endothelial model of monogenic diabetes**

*Dawid Skoczek, Damian Kloska, Marta Targosz-Korecka, Krzysztof Szade, Artur Biela, Jerzy Hohendorff, Marian Babincak, Aleksandra Kopacz, Maciej T. Malecki, Jacek Stepniewski, Neli Kachamakova-Trojanowska^*^*

**Supplementary Methods:**

**Sanger sequencing**

To identify or confirm the nature and exact location of mutations in *HNF1A*, Sanger sequencing was performed. Lineage-specific genomic DNA was isolated using the GenomicMini DNA Extraction Kit (A&A Biotechnology). Subsequently, 50 ng of lineage-specific genomic DNA was used as template to amplify an approximately 650-nucleotide fragment encompassing the mutation-containing *HNF1A* genomic region by PCR using HiFi Polymerase (TaKaRa Bio) on a ProFlex PCR System (Thermo Fisher Scientific). The resulting PCR products were purified and submitted for Sanger sequencing. Sequence analysis was performed using SnapGene Viewer software to confirm the presence and precise location of mutations within the *HNF1A* gene. The sequences of primers used are listed in Table 1.

Table 1. List of primers used for the identification of the HNF1A mutations

| Line | Sequence |
| --- | --- |
| MAC & BAC | F: CCTTTATCTGTTCCAGTGTCTGT  R: CAGGACCAAGTCTACTCCCGTC |
| MP_1 | F: ATGGTTTCTAAACTGAGCCAGC  R: CAGTTCTCCAGGCATTTGAGTT |
| MP_2 | F: CTTGAACCTAAGCCCATTCCTC  R: GAAGTGCAAAGAGGTTTAGGTG |

**Verification of absence of Sendai Virus in hiPSC lines using RT-PCR and PCR analysis**

To confirm the absence of Sendai virus components in hiPSC lines, we performed RT-PCR and PCR analyses. Total RNA was extracted from hiPSCs using the Total RNA Mini Kit (A&A Biotechnology) according to the manufacturer's protocol. For reverse transcription, 1 µg of total RNA was converted to cDNA using the High-Capacity cDNA Reverse Transcription Kit (Thermo Fisher Scientific) in a ProFlex PCR System (Thermo Fisher Scientific). PCR amplification was performed using 50 ng of cDNA template, gene-specific primers (Table 2), and KAPA2G Fast Genotyping Mix (Merck) in the ProFlex PCR System. The PCR products were resolved by electrophoresis on 2% agarose gel.

Table 2. List of primers used for the identification of Sendai vectors

| Target | Sequence |
| --- | --- |
| eEF2  (House-keeping gene) | F: TGAGCACACTGGCATAGAGGC  R: GACATCACCAAGGGTGTGCAG |
| Sendai Vectors | F: GGATCACTAGGTGATATCGAGC  R: ACCAGACAAGAGTTTAAGAGATATGTATC |

**Spontaneous *in vitro* differentiation of hiPSC lines via embryoid bodies (EBs)**

To evaluate the pluripotent differentiation capacity of hiPSC lines, we performed embryoid body (EB) formation assays. hiPSCs were harvested with 0.5 mM EDTA and seeded at 4,000 cells per well in non-adherent U-shaped 96-well plates (Corning) in Essential 6 medium (E6, Gibco) supplemented with 10 µM Y27632 (Focus Biomolecules) and 4 mg/mL polyvinyl alcohol (Merck). EBs were cultured for 7 days with medium changes every 48 hours. After initial EB formation, spheroids were transferred to Geltrex-coated 48-well plates with fresh E6 medium and cultured for an additional 3 weeks with medium changes every 2-3 days. Differentiated EBs were then analyzed for lineage-specific markers representing all three germ layers.

**Immunofluorescent analysis**

**Pluripotency marker assessment**

For pluripotency marker evaluation, hiPSCs were seeded at 2.0 × 10³ cells per well in Geltrex-coated 48-well plates in Essential 8 Medium (E8, Gibco) supplemented with 10 μM Y-27632 (Focus Biomolecules). After 24 hours, the medium was replaced with E8 alone, and cells were cultured for an additional 24 hours before immunostaining.

**hiPSC-ECs characterization**

hiPSC-derived endothelial cells (hiPSC-ECs) were seeded at 3.0 × 10⁴ cells per coverslip on fibronectin-coated (Gibco) 13 mm coverslips in 24-well plates. Cells were cultured in EGM-MV2 medium (Promo Cell) supplemented with 50 ng/mL VEGF (StemCell Technology) for 48 hours before immunostaining.

**Immunostaining protocol**

All immunofluorescence procedures followed a standardized protocol. Cells were washed twice with PBS and fixed with 4% paraformaldehyde (Sigma) for 15 minutes at room temperature (RT). After washing with PBS, cells were permeabilized with 0.1% Triton X-100 in PBS for 15 minutes at RT. Cells were then incubated in blocking buffer (5% bovine serum albumin in PBS, Sigma-Aldrich) for 1 hour at RT.

For primary antibody incubation, cells were incubated overnight at 4°C with primary antibodies diluted in blocking buffer. Pluripotency markers included anti-Oct-3/4, anti-Nanog, anti-SSEA-4, and anti-TRA-1-60. Germ layer markers consisted of anti-GATA-4 for endoderm, anti-Vimentin for mesoderm, and anti-Tubulin-β3 for ectoderm. Endothelial marker assessment utilized anti-VE-Cadherin antibody.

After washing three times with PBS, cells were incubated with appropriate secondary antibodies for 1 hour at RT. Nuclei were counterstained with DAPI (4′,6-diamidino-2-phenylindole) for 10 minutes at RT. Fluorescence images were captured using a Leica DMi8 microscope. A detailed list of antibodies and their corresponding dilutions used for immunofluorescence staining is provided in Table 3.

Table 3. List of used antibodies and dyes

| Antibody/Dye | Type | Dilution/ concentration | Catalog number | Company |
| --- | --- | --- | --- | --- |
| Goat anti-Oct 3/4 | Primary | 1:200 | sc-8628 | Santa Cruz Biotechnology |
| Mouse anti-Nanog | Primary | 1:100 | sc-293121 | Santa Cruz Biotechnology |
| Mouse anti-SSEA4 | Primary | 1:100 | sc-21704 | Santa Cruz Biotechnology |
| Mouse anti-TRA-1-60 | Primary | 1:100 | SCR001 | Sigma-Aldrich |
| Mouse GATA-4 | Primary | 1:200 | sc-25310 | Santa Cruz Biotechnology |
| Rabbit Vimentin | Primary | 1:200 | MA5-35320 | Invitrogen |
| Mouse Tubulin-β3 | Primary | 1:200 | 801213 | BioLegend |
| Mouse VE-Cadherin | Primary | 1:200 | GTX633705 | GeneTex |
| Donkey anti-goat labeled with Alexa-Fluor 488 | Secondary | 1:500 | A32814 | Thermo Fisher Scientific |
| Donkey anti-goat labeled with Alexa-Fluor 488 | Secondary | 1:500 | A21206 | Thermo Fisher Scientific |
| Donkey anti-mouse labeled with Alexa-Fluor 568 | Secondary | 1:500 | A10037 | Thermo Fisher Scientific |
| DAPI | DNA dye | 0,2 µg/ml | D9542 | Sigma-Aldrich |

**Karyotype genetic test**

hiPSCs were cultured in vitro under standard conditions. Following karyotype maintenance procedures, chromosome preparations were subjected to GTG-banding at the 450-band level. Metaphase spreads were analyzed using the GenASIs imaging platform (Applied Spectral Imaging). Karyotype description was performed in accordance with the International System for Human Cytogenomic Nomenclature (ISCN 2020). All cytogenetic analyses were carried out by certified specialists at Karyogen, Kraków, Poland.

**Flow cytometry analysis**

Flow cytometry was performed to quantify endothelial-specific marker expression in hiPSC-ECs. Cells were seeded at 6.0 × 10⁴ cells per well in 12-well plates and cultured in EGM-MV2 medium (Promo Cell) supplemented with 50 ng/mL VEGF (StemCell Technology). After 48 hours, cells were dissociated using Accutase (Gibco) and centrifuged at 200 g for 5 minutes at RT. Cell pellets were resuspended and incubated with fluorophore-conjugated antibodies diluted 1:100 in 2% FBS-PBS for 25 minutes at 4°C, specifically PE Mouse Anti-Human CD31 (Cat: 555446, BD Biosciences) and Alexa Fluor 647 Mouse Anti-Human CD144 (Cat: 561567, BD Biosciences). After washing with PBS, cells were resuspended in PBS containing 0.2 µg/mL DAPI for viability assessment. Data acquisition was performed using an LSRFortessa cytometer, and analysis was conducted with BD FACSDiva™ software (BD Biosciences). Flow cytometry data were analyzed using appropriate gating strategies to exclude debris and dead cells. Positive expression was determined using unstained and single-color controls to establish compensation and gating parameters.

**Predicted structural models of wild-type HNF1A and HNF1A mutations**

Structural predictions for wild-type HNF1A and HNF1A mutations present in HNF1A-MODY patients (MP_1 and MP_2) were generated using the *Homo sapiens* HNF1A CCDS 9209.1 sequence. Three-dimensional protein structure models were predicted using AlphaFold3.0 [1] to generate homodimeric and heterodimeric configurations, including wild-type HNF1A homodimers, wild-type/mutant heterodimers (WT-MP1 and WT-MP2), and mutant homodimers (MP2-MP2). Additional models were generated with bound double-stranded HNF1A-specific inverted palindromic DNA sequence (5'-GTTAATNATTAAC-3') to assess protein-DNA interactions. Structural predictions were colored according to the prediction confidence score (pIDDT), and unstructured regions were hidden to enhance visualization clarity. All structural figures were prepared and visualized using ChimeraX software [2].

**References:**

1. Abramson J, Adler J, Dunger J, Evans R, Green T, Pritzel A, et al. Accurate structure prediction of biomolecular interactions with AlphaFold 3. Nature. 2024;630:493–500.

2. Meng EC, Goddard TD, Pettersen EF, Couch GS, Pearson ZJ, Morris JH, et al. UCSF ChimeraX: Tools for structure building and analysis. Protein Sci. 2023;32:e4792.
